# Tolerability, safety, and immunogenicity of the novel oral polio vaccine type 2 in children aged 6 weeks to 59 months in an outbreak response campaign in The Gambia: an observational cohort study

**DOI:** 10.1016/S1473-3099(23)00631-X

**Published:** 2024-04

**Authors:** Adedapo O Bashorun, Larry Kotei, Ousubie Jawla, Abdoulie F Jallow, Aisha J Saidy, Ma-Ansu Kinteh, Arafang Kujabi, Tijan Jobarteh, Francis John Kanu, Simon A Donkor, Esu Ezeani, Sidat Fofana, Mbye Njie, Lamin Ceesay, Basit Jafri, Amanda Williams, David Jeffries, Brezesky Kotanmi, Bernardo A Mainou, Michael Ooko, Ed Clarke

**Affiliations:** a MRC Unit The Gambia at the London School of Hygiene & Tropical Medicine, Banjul, The Gambia; b Expanded Programme on Immunization, Ministry of Health, Government of The Gambia, Kotu, The Gambia; c Division of Viral Diseases, National Center for Immunization and Respiratory Diseases, US Centers for Disease Control and Prevention, Atlanta, GA, USA

## Abstract

**Background:**

Novel oral polio vaccine type 2 (nOPV2) has been used to interrupt circulating vaccine-derived poliovirus type 2 outbreaks following its WHO emergency use listing. This study reports data on the safety and immunogenicity of nOPV2 over two rounds of a campaign in The Gambia.

**Methods:**

This observational cohort study collected baseline symptoms (vomiting, diarrhoea, irritability, reduced feeding, and reduced activity) and axillary temperature from children aged 6 weeks to 59 months in The Gambia before a series of two rounds of a nOPV2 campaign that took place on Nov 20–26, 2021, and March 19–22, 2022. Serum and stool samples were collected from a subset of the participants. The same symptoms were re-assessed during the week following each dose of nOPV2. Stool samples were collected on days 7 and 28, and serum was collected on day 28 following each dose. Adverse events, including adverse events of special interest, were documented for 28 days after each campaign round. Serum neutralising antibodies were measured by microneutralisation assay, and stool poliovirus excretion was measured by real-time RT-PCR.

**Findings:**

Of the 5635 children eligible for the study, 5504 (97·7%) received at least one dose of nOPV2. There was no increase in axillary temperature or in any of the baseline symptoms following either rounds of the campaigns. There were no adverse events of special interest and no other safety signals of concern. Poliovirus type 2 seroconversion rates were 70% (95% CI 62 to 78; 87 of 124 children) following one dose of nOPV2 and 91% (85 to 95; 113 of 124 children) following two doses. Poliovirus excretion on day 7 was lower after the second round (162 of 459 samples; 35·3%, 95% CI 31·1 to 39·8) than after the first round (292 of 658 samples; 44·4%, 40·6 to 48·2) of the campaign (difference –9·1%; 95% CI –14·8 to –3·3), showing the induction of mucosal immunity.

**Interpretation:**

In a campaign in west Africa, nOPV2 was well tolerated and safe. High rates of seroconversion and evidence of mucosal immunity support the licensure and WHO prequalification of this vaccine.

**Funding:**

Bill & Melinda Gates Foundation.

## Introduction

Although the goal of polio eradication is within reach, hurdles remain. Wild poliovirus types 2 and 3 were certified as eradicated in 2015 and 2019, respectively. Only Pakistan and Afghanistan remain endemic for wild poliovirus type 1 and reported a total of 20 cases of paralytic disease in the 12 months before April 18, 2023.^1^

Oral polio vaccines (OPVs) remain crucial to the eradication strategy. These vaccines not only generate systemic antibodies, but also stimulate mucosal immunity, reducing excretion of the virus and hence community transmission.^2^ However, a disadvantage of OPVs is the emergence of circulating vaccine-derived polioviruses (cVDPVs) that are genetically divergent from the parent strain—having regained the capacity for person-to-person transmission similar to wild polioviruses—and occasionally cause paralytic disease, reflecting reversion to neurovirulence.^3^ In April, 2016, a switch from trivalent OPVs (tOPVs) to bivalent OPVs (bOPVs), which contain only the Sabin type 1 and 3 strains, was made in all OPV-using countries globally. The switch aimed to reduce the occurrence of cVDPVs, over 85% of which were attributable to the type 2 vaccine virus at the time. However, it has also resulted in a marked global decrease in type 2 mucosal immunity.^3^, ^4^ Consequently, although there were fewer than 100 cases of circulating vaccine-derived poliovirus type 2 (cVDPV2) in two countries in 2017, there was a sustained increase in the number of cases in the following years, with over 1000 cases being detected in 24 countries by 2020.^5^ The detection of cVDPV2 in Europe, North America, and southeast Asia emphasises the global nature of the threat posed by cVDPVs. Given the necessity to block transmission, Sabin monovalent OPV type 2 (mOPV2) immunisation campaigns have been the predominant method of outbreak response for cVDPV2. Consequently, although early outbreaks were seeded from the use of tOPV before the switch, sequencing data confirm that, since as early as 2017, cVDPV2 outbreaks have increasingly arisen from the administration of mOPV2 in such campaigns.^6^

**Research in context**

**Evidence before this study**

A PubMed search to identify articles published before June 18, 2023, was conducted with the following search terms with appropriate Boolean operators: “oral poliovirus vaccine”, “vaccine derived poliovirus”, “campaign”, “meta-analysis”, “systematic review”, “randomized controlled trial”, “clinical trial”, “immunogenicity”, “safety”, and “mucosal immunity”.

Novel oral polio vaccine type 2 (nOPV2) has been engineered to increase the genetic stability of type 2 Sabin OPV and, therefore, decrease outbreaks of circulating vaccine-derived poliovirus type 2 (cVDPV2). In clinical trials conducted in adults in Belgium and in toddlers and infants in Panama, the vaccine has been shown to be well tolerated and safe. In Panama, 86–94% of children had an immune response following one dose of nOPV2 and at least 98% of children had an immune response following two doses of the vaccine. nOPV2 has been shown to generate mucosal immunity and to have a lower risk of reversion to neurovirulence than type 2 Sabin OPV. On the basis of these data, over 500 million doses of the vaccine have subsequently been administered across 23 countries without notable safety signals. Following a cVDPV2 outbreak response campaign in Tajikistan in central Asia, seroconversion rates of 67% following one dose and 77% following two doses have been reported. However, following two nOPV2 campaign rounds in Liberia in west Africa, the type 2 seroprevalence was 38%.

**Added value of this study**

This study shows that over the course of two rounds of an nOPV2 outbreak response campaign in The Gambia, the vaccine was found to be safe and well tolerated. There was no increase in the occurrence of any common symptoms following either round of the campaign and there were no adverse events of special interest in relation to nOPV2. Overall, 70% of type 2 seronegative children seroconverted following one dose of nOPV2, and 91% of these children seroconverted following two doses. The post-campaign type 2 seroprevalence in children who had received two doses of nOPV2 was 97%. The vaccine generated mucosal immunity in the Gambian childhood population.

**Implications of all the available evidence**

nOPV2 is well tolerated and safe in both clinical trials and outbreak response campaigns. Data on the immunogenicity of the vaccine is heterogeneous, although the majority of data suggest the vaccine has similar immunogenicity to Sabin OPV. The vaccine induces intestinal mucosal immunity and is therefore expected to interrupt community transmission of cVDPV2. The data support the licensure and WHO prequalification of nOPV2, although further immunogenicity studies of the vaccine from diverse settings (particularly when used in campaigns) would be valuable.

The novel OPV type 2 (nOPV2) strain was engineered to improve the genetic stability of the Sabin type 2 OPV, and therefore reduce the occurrence of cVDPV2 and the incidence of vaccine-associated paralytic poliomyelitis.^7^ On the basis of supportive preclinical data, the vaccine was tested in phase 1 and 2 trials in adults in Belgium between 2017 and 2019 and in phase 2 trials in children aged 1–4 years and infants aged 18–22 weeks in Panama from 2018 to 2019.^7^, ^8^, ^9^, ^10^ In all three age groups, nOPV2 was well tolerated and safe. Serological responses to the vaccine were similar to those generated in historical mOPV2 controls. The vaccine was shown to generate mucosal immunity. Neurovirulence testing also suggested a reduced risk of reversion to virulence following nOPV2 immunisation compared with mOPV2 immunisation.^11^ Next generation sequencing has subsequently confirmed the enhanced genotypic stability of the new strain.^11^

On the basis of these data (and due to the rapid increase in outbreaks of cVDPV2, and their status as a Public Health Emergency of International Concern) in November, 2020, nOPV2 was the first vaccine globally to be given emergency use listing by WHO.^12^ The vaccine was used as part of a cVDPV2 outbreak response campaign in Nigeria in March, 2021, and over 800 million doses of the vaccine have now been administered through campaigns across 28 countries.^12^

This observational cohort study reports the first comprehensive set of data on the tolerability, safety, and systemic and mucosal immunogenicity of nOPV2 when administered as part of an outbreak response campaign. The campaign was undertaken in The Gambia in west Africa, in response to the detection of cVDPV2 in environmental surveillance samples.

## Methods

### Study design and setting

This observational cohort study was conducted across Sibanor, Soma, and Basse—three rural and periurban sites in The Gambia. It was approved by the Gambian Government/Medical Research Council Joint Ethics Committee and the London School of Hygiene & Tropical Medicine Research Ethics Committee (LEO 26472). All children aged 6 weeks to 59 months living in the study areas whose parents provided informed written consent were eligible for participation.

4–6 weeks before the first round of the campaign, demographic information and vaccination history (from parent-held record cards) were collected from participants (appendix p 1). Axillary temperatures were measured with calibrated thermometers. Weight-for-length Z scores were calculated on the basis of WHO reference ranges. Baseline data on the presence and severity of vomiting, diarrhoea, irritability, reduction in feeding, and reduction in activity (compared with normal behaviour) on the given day were collected by trained fieldworkers (appendix p 2). Baseline serum and stool samples were collected from a subset of the participants in Soma for logistical reasons. The participants in Soma share similar sociodemographic characteristics to the background population.

Axillary temperature and data on the same set of symptoms were collected from each participant on 1 day distributed over the course of the first week following each of the two rounds of the nOPV2 campaign. To maximise consistency, the same field workers collected the data at baseline and at the post-campaign follow-up visits. Parents were also asked to contact the study team if their child had any illness over the 4 weeks following each round of the campaign. Unsolicited adverse events following immunisation were documented and specific inquiries were made to ensure that adverse events of special interest (AESIs; appendix pp 3–5) were recorded. Parents were also contacted 4 weeks after each round of the campaign to ensure all adverse events had been recorded. Serum samples were collected from the subset of participants in Soma on day 28, and stool samples were collected on days 7 and 28 following each round of the campaign.

Serum and stool samples were frozen at below –70°C before being shipped to the US Centers for Disease Control and Prevention laboratories (Atlanta, GA, USA; appendix p 6). Serotype-specific poliovirus serum neutralising antibodies (SNAs) were measured with the WHO standard microneutralisation assay (WHO EPI GEN 93.9), adapted as previously described.^13^ The lower and upper limits of quantification for reciprocal SNA titres were a log_2_ titre less than 2·5 and a log_2_ titre greater than 10·5, respectively. Serotype-specific polioviruses were detected in stool samples with a validated real-time RT-PCR (rtRT-PCR) assay.^14^ A nOPV2-specific rtRT-PCR was conducted for those samples that were positive for a type 2 virus at baseline.

### Outcomes

The severity of any vomiting, diarrhoea, irritability, reduction in feeding, and reduction in activity was defined according to the Division of AIDS tables (appendix p 2).^15^ Adverse events following immunisation were coded by the preferred term according to the Medical Dictionary of Regulatory Activities (version 25.1).

The immunogenicity endpoints were seroprotection rates (percentage of participants with a reciprocal SNA titre ≥8 [3log_2_]), seroconversion rates (percentage of participants with a baseline reciprocal SNA titre <8 and a post-vaccination reciprocal SNA titre ≥8), rates of 4-fold titre rise (percentage of participants with a baseline reciprocal log_2_SNA titre of ≥8 and ≤362 [8·5log_2_] who had a 4-fold rise in reciprocal SNA titres following vaccination), immune response rates (combining the number of participants undergoing seroconversion with the number experiencing a 4-fold titre rise), median log_2_ antibody titres, geometric mean titres, geometric mean fold rise in titres from baseline, and the percentage of participants excreting serotype-specific polioviruses at each of the timepoints. Participants who received routine doses of inactivated polio vaccine (IPV) or bOPV after the baseline samples were collected but before the post-campaign samples were collected were excluded from the serological and stool analyses.

### Statistical analysis

The study was designed to provide estimates for the rates of common safety events following vaccination with sufficient precision to support submissions for nOPV2 licensure and WHO prequalification and to guide policy decisions, and with a high probability of detecting less frequent safety events (appendix p 7). The analysis is descriptive and 95% CIs are used throughout. CIs around proportions and differences in independent proportions were calculated with the Wilson score methods.^16^, ^17^ CIs around differences in paired axillary temperatures were calculated with a paired *t* test, and CIs around proportions of solicited adverse events were calculated with Bonett and Price's adjusted wald for paired proportions.^18^ CIs around unsolicited adverse event rates were calculated with the Wilson score interval when children had up to one of a given event. Bootstrap CIs were calculated when participants had more than one of a given event. CIs around geometric mean titres and geometric mean fold change were calculated with Efron's non-parametric, bias-corrected, and accelerated bootstrap method.^19^ SNA titres of less than 2·5 were taken as 1·25 and titres of more than 10·5 were taken as 10·5 for quantitative analysis. Statistical analyses were done with R (version 4.2.2).

### Role of the funding source

The study funder provided advice on the study design and data interpretation.

## Results

**Figure 1:**
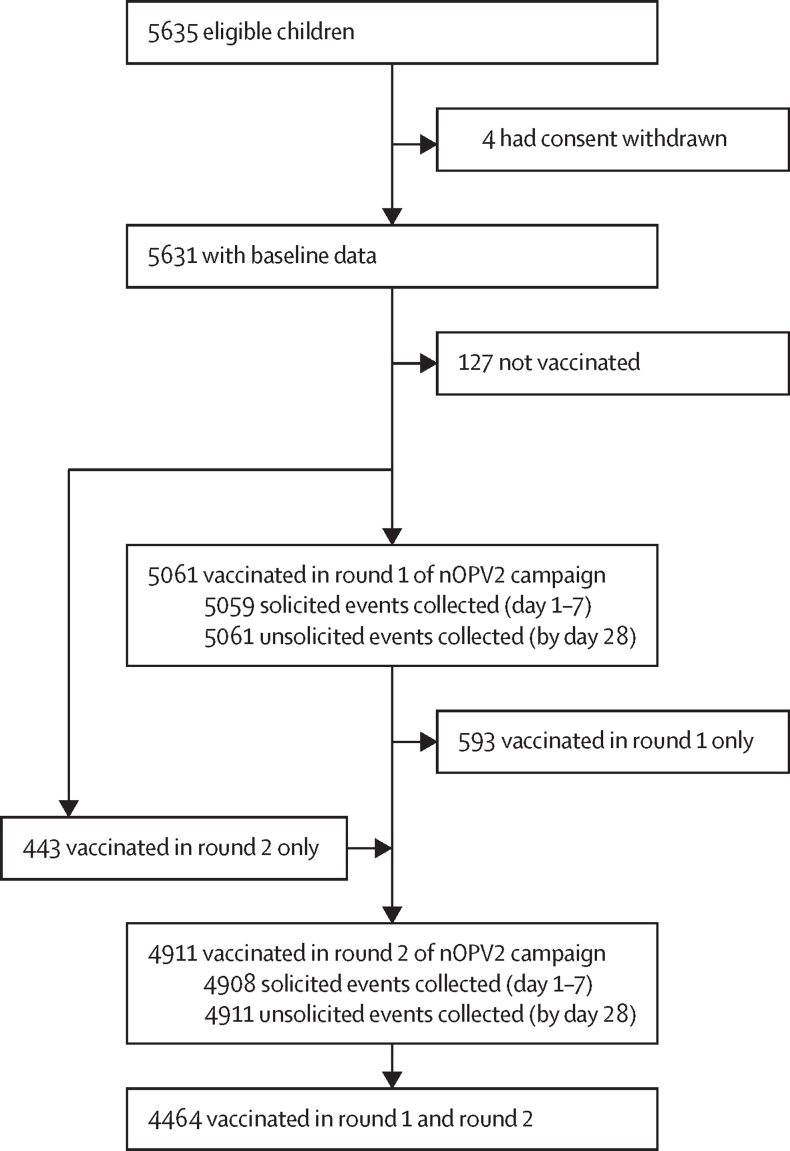
Study profile nOPV2=novel oral polio vaccine type 2.

From Oct 5 to Oct 31, 2021, informed consent was obtained from the parents of 5635 children aged 6 weeks to 59 months (figure 1). The first round of the campaign took place between Nov 20 and Nov 26, 2021, and the second round between March 19 and March 22, 2022. Of the children for whom consent was obtained, 5504 (97·7%) were reported to have received at least one dose of nOPV2 over the two rounds of the campaign (5061 [89·9%] of 5635 during round 1 and 4911 [87·2%] of 5635 during round 2). Solicited adverse event data were collected from more than 99·9% of vaccinated children (5059 of 5061 after round 1, and 4908 of 4911 after round 2), all of whom had safety data collected up to day 28. 371 participants had serum samples collected and SNA results available at baseline, post round 1, and post round 2.

Demographic, anthropometric, and polio vaccination data on the population who received at least one dose of nOPV2 are provided in table 1. There was an equal split of male and female participants, of whom 42·8% (2356 of 5504) were younger than 1 year. Overall, 6·7% of participants (364 of 5453) were severely malnourished. Before the start of the campaign, over 92% (5073 of 5504) of participants had received more than 3 doses of bOPV. Over 82% (4345 of 5247) of participants had received at least one dose of IPV.

**Table 1:** Demographic, anthropometric, and vaccination history of vaccinated participants

		Vaccinated participants*
Sex†		
	Male	2745/5504 (49·9%)
	Female	2759/5504 (50·1%)
Age at consent		
	≤1 year	2356/5504 (42·8%)
	>1 year to ≤2 years	1256/5504 (22·8%)
	>2 years to ≤3 years	1046/5504 (19·0%)
	>3 years to ≤4 years	812/5504 (14·8%)
	>4 years	34/5504 (0·6%)
Age at vaccination 1, years		2·3 (1·3–3·5)
Age at vaccination 2, years		2·7 (1·6–3·8)
Ethnic group		
	Mandinka	2404/5504 (43·7%)
	Fula	1813/5504 (32·9%)
	Wollof	469/5504 (8·5%)
	Jola	422/5504 (7·7%)
	Other	396/5504 (7·2%)
Number of siblings		2 (1–4)
Occupation of mother		
	Employed outside the home	1819/5504 (33·0%)
	Not employed outside the home	3683/5504 (66·9%)
	Other	2 (<0·1%)
Number of years of English school completed by mother		
	0	3525/5504 (64·0%)
	>0	1979/5504 (36·0%)
Birthweight, kg		3·0 (0·5)
Weight-for-length Z score		
	Z <–3 (severe malnourishment)	364/5453 (6·7%)
	−3 ≤Z ≤–2 (moderate malnourishment)	623/5453 (11·4%)
	Z >–2	4466/5453 (81·9%)
Number of bivalent OPV doses received before the campaign‡		
	0	22/5504 (0·4%)
	1–3	409/5504 (7·4%)
	>3	5073/5504 (92·2%)
Inactivated polio vaccine received before the campaign		
	Yes	4345/5247 (82·8%)
	No	902/5247 (17·2%)
Number of vitamin A doses received before the campaign		2 (1–3)
Number of mebendazole doses received before the campaign		2 (1–3)

Data are n/N (%), median (IQR), or mean (SD). Data were collected at the time of enrolment, before either round of the campaign. OPV=oral poliovirus vaccine. OPV2=oral polio vaccine type 2.

* Participants who received at least one dose of novel OPV2 over the two rounds of the campaign.

† Based on parental reports.

‡ None of the participants had received trivalent OPV or monovalent OPV2 before this study.

**Figure 2:**
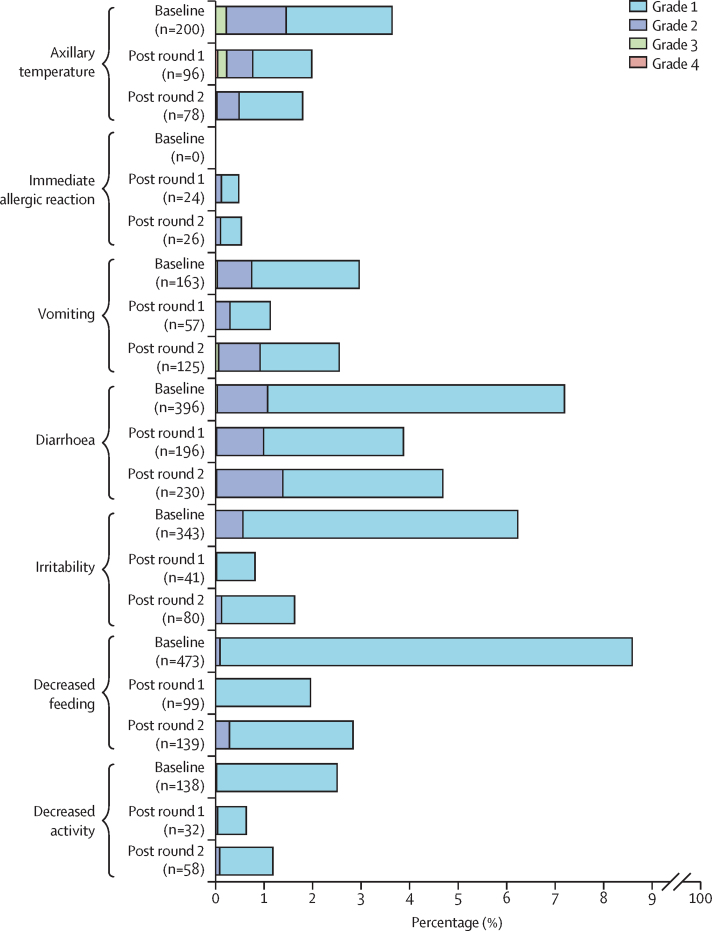
Proportion of participants with baseline and solicited adverse events in the 7 days following a first and second dose of nOPV2 Percentage calculated as n/N (where N=5631 at baseline, N=5061 after round 1, and N=4911 after round 2). nOPV2=novel oral polio vaccine type 2.

There was no increase in any solicited symptom over the first week following either the first or the second round of the campaign compared with the baseline (figure 2; appendix pp 8–9). The mean axillary temperature in participants was lower following vaccination (36·27°C [95% CI 36·25–36·29] post dose 1 and 36·22°C [36·20–36·24] post dose 2) than at baseline (36·38°C [36·36–36·40]), although the difference is not clinically meaningful. Two participants had a temperature of greater than 40·5°C recorded following the first round of the campaign. Both had associated diarrhoea and made a full recovery. The percentage of parents reporting vomiting, diarrhoea, irritability, or reductions in either feeding or activity level tended to be lower in the weeks following each round of the campaign than at baseline. There was no apparent temporal association in the frequency of any symptom comparing children from whom data were collected within the first 2 days after administration of nOPV2 with those from whom data were collected later in the first week (appendix pp 10–13). Other than a marginal increase (1·01%, 95% CI 0·03–1·99) in vomiting following the second round of the campaign in children from non-Mandinka ethnic groups—of unlikely clinical significance in isolation—there were no other increases in the frequency of symptoms on stratified analysis (appendix pp 14–23).

There were no AESIs in children enrolled in the study (table 2). Following the first round of the campaign, 380 adverse events occurred in 322 (6·4%) of 5061 children. Following the second round, 804 adverse events occurred in 624 (12·7%) of 4911 children. The increase in the number of events following the second round of the campaign reflected an increase in the number of mild events, which were predominantly respiratory and gastrointestinal complaints (appendix pp 24–27). There was no change in the occurrence of moderate and severe events (116 of 5061 children [2·3%, 95% CI 1·9–2·7] in round 1 and 89 of 4911 children [1·8%, 1·5–2·2] in round 2) and there were no consistent temporal trends to suggest causality (appendix p 28).

**Table 2:** Unsolicited adverse events in vaccinated participants including adverse events of special interest

		**Post round 1 (N=5061)**		**Post round 2 (N=4911)**	
		Children with an event (%)	Total events	Children with an event (%)	Total events
Any adverse event		322 (6·4%)	380	624 (12·7%)	804
Severity of adverse events*					
	Mild	206 (4·1%)	250	535 (10·9%)	708
	Moderate	105 (2·1%)	117	83 (1·7%)	89
	Severe	11 (0·2%)	13	6 (0·1%)	7
Serious adverse events		5 (0·1%)	7	6 (0·1%)	8
	Life-threatening adverse events	0	0	2 (<0·1%)	2
	Admitted to hospital	5 (0·1%)	7	5 (0·1%)	7
	New long-term disability	0	0	0	0
	Death	0	0	1 (<0·1%)	1
Related serious adverse events		0	0	0	0
Adverse events of special interest		0	0	0	0

N=total number of participants for whom safety data were collected post round 1 and post round 2 of the campaign.

* Maximum severity of any event in a given participant.

There were seven serious adverse events in five children (0·1%, N=5061) following the first round of the campaign and eight serious adverse events in six children (0·1%, N=4911) following the second round. One child died with a diagnosis of gastroenteritis (appendix p 29). None of the serious adverse events were judged to be related to nOPV2.

**Figure 3:**
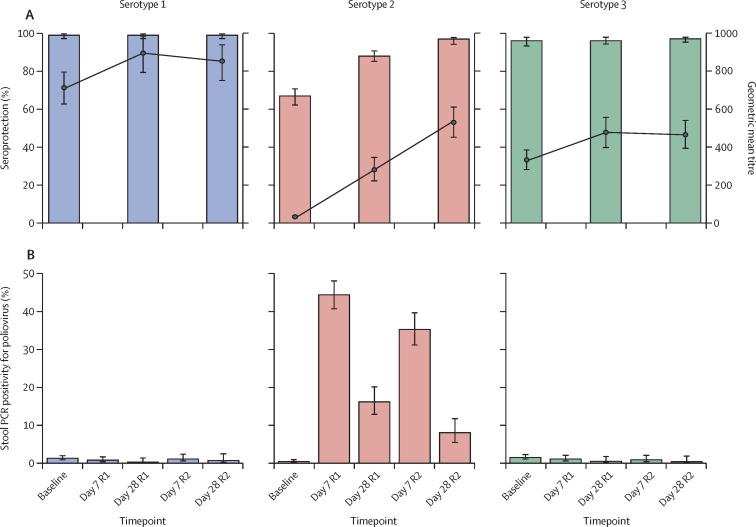
Seroprotection rates and geometric mean serum neutralising antibody titres (A), and stool PCR positivity for poliovirus (B) at baseline and R1 and R2 of the nOPV2 campaign (A) Bars indicate serotype-specific seroprotection rates (left y-axis); points indicate serotype-specific geometric mean serum neutralising antibody titres (right y-axis). Seroprotection rate is defined as the percentage of participants with a serum neutralising antibody titre of 8 or more. (B) Stool real-time RT-PCR positivity for serotype-specific poliovirus. Error bars show 95% CIs. nOPV2=novel oral polio vaccine type 2. R1=following round 1 of the campaign. R2=following round 2 of the campaign.

Poliovirus type 2 seroprotection rates were 67% (95% CI 62–71; 247 of 371 children) at baseline, increasing to 88% (85–91; 328 of 371) following the first round of the campaign and 97% (94–98; 359 of 371) following the second round (table 3, figure 3A). The serotype 2 seroconversion rate, in children who were seronegative at baseline, was 70% (95% CI 62–78; 87 of 124 children) following one dose of nOPV2 and 91% (85–95; 113 of 124) following two doses. The serotype 2 immune response rate following one dose of nOPV2 was 66% (95% CI 60–71; 199 of 303 children) and 86% (82–90; 261 of 303) following two doses. A third of children in the study (25 of 75 for serotype 1 and 51 of 155 for serotype 3) had an immune response to poliovirus serotypes 1 and 3 over the two rounds of the campaign (table 3, figure 3A). SNA reverse cumulative distribution curves are provided (appendix p 30).

**Table 3:** Serotype-specific poliovirus neutralising antibody responses at baseline, post round 1, and post round 2 of the nOPV2 campaign

	**Serotype 1**	**Serotype 2**	**Serotype 3**
**Baseline**			
Median	10·17 (8·83–10·50)	4·83 (1·25–7·17)	8·83 (7·50–10·17)
Geometric mean titre	713·65 (625·30–798·25)	32·66 (26·01–41·30)	332·22 (278·79–387·11)
Seroprotection	99% (327/331; 97–100)	67% (247/371; 62–71)	96% (318/331; 93–98)
**Post round 1**			
Median	10·50 (9·83–10·50)	10·17 (5·83–10·50)	9·83 (8·17–10·50)
Geometric mean titre	895·95 (791·62–987·65)	280·62 (219·69–347·96)	477·20 (395·17–558·51)
Seroprotection	99% (327/331, 97–100)	88% (328/371, 85–91)	96% (319/331, 94–98)
Seroconversion (baseline seronegative)	0% (0/4, 0–49)	70% (87/124, 62–78)	8% (1/13, 1–33)
4-fold rise (baseline seropositive)	42% (30/71, 31–54)	63% (112/179, 55–69)	35% (49/142, 27–43)
Immune response (overall)	40% (30/75, 30–51)	66% (199/303, 60–71)	32% (50/155, 25–40)
Geometric mean fold rise (post round 1 *vs* baseline)	1·26 (1·06–1·49)	8·59 (6·13–11·81)	1·44 (1·13–1·83)
**Post round 2**			
Median	10·50 (9·50–10·50)	10·50 (8·17–10·50)	9·83 (7·83–10·50)
Geometric mean titre	853·90 (749·58–942·25)	530·63 (450·01–613·6)	464·68 (390·75–542·68)
Seroprotection	99% (328/331, 97–100)	97% (359/371, 94–98)	97% (321/331, 95–98)
Seroconversion (baseline seronegative)	25% (1/4, 5–70)	91% (113/124, 85–95)	23% (3/13, 8–50)
4-fold rise (baseline seropositive)	34% (24/71, 24–45)	83% (148/179, 76–88)	34% (48/142, 27–42)
Immune response (overall)	33% (25/75, 24–45)	86% (261/303, 82–90)	33% (51/155, 26–41)
Geometric mean fold rise (post round 2/baseline)	1·20 (1·02–1·41)	16·25 (12·22–21·21)	1·40 (1·11–1·77)
Seroconversion (seronegative post round 1)	25% (1/4, 5–70)	79% (34/43, 65–89)	17% (2/12, 5–45)
4-fold rise (seropositive post round 1)	31% (12/39, 19–46)	68% (63/93, 58–76)	32% (31/97, 24–42)
Immune response (overall post round 1)	30% (13/43, 19–45)	71% (97/136, 63–78)	30% (33/109, 22–39)
Geometric mean fold rise (post round 2/post round 1)	0·95 (0·82–1·12)	1·89 (1·43–2·49)	0·97 (0·77–1·23)

Data are median (IQR), mean (95% CI), and % (n/N, 95% CI). Seroprotection is defined as the percentage of children with a serotype-specific neutralising antibody titre of 8 or more. Seroconversion is defined as the percentage of children with a serotype-specific neutralising antibody titre of 8 or more post vaccination among those with a serotype-specific neutralising antibody titre of less than 8 prevaccination. 4-fold rise is defined as the percentage of children experiencing a 4-fold rise in serotype-specific neutralising antibody titres post vaccination among those with a serotype-specific neutralising antibody titre of 8 or more prevaccination. Immune response combines the number of children undergoing seroconversion with those having a 4-fold rise in titres. n/N=Number of participants with indicated immune response/number of participants available for given assessment. nOPV2=novel oral polio vaccine type 2.

At baseline, 0·5% (95% CI 0·2 to 1·1; 6 of 1162) of participants had a non-nOPV2 type 2 poliovirus detectable in their stool (figure 3B; appendix p 31). On day 7 following administration of nOPV2, 44·4% (95% CI 40·6 to 48·2; 292 of 658) of participants were excreting the virus following round 1 compared with 35·5% (31·1 to 39·8; 162 of 459) following round 2 (difference –9·1%; 95% CI –14·8 to –3·3). On day 28, the equivalent percentages were 16·2% (95% CI 12·8 to 20·3; 61 of 376) following round 1 and 8·1% (5·4 to 11·9**;** 22 of 272) following round 2 (difference –8·1%; 95% CI –13·1 to –3·1). The more rapid clearance of the virus at both timepoints supports the induction of mucosal immunity by nOPV2.

## Discussion

This observational cohort study provides a comprehensive set of data on the tolerability, safety, and systemic and mucosal immunogenicity of nOPV2 administered in a cVDPV2 outbreak response campaign. The data complement clinical trial results and are part of the package submitted to WHO to support prequalification of the vaccine.^8^, ^9^, ^10^, ^20^ The findings are also of value to national public authorities preparing to use nOPV2.

The vaccine was safe and well tolerated in this study, irrespective of age and nutritional status. No neurological or severe allergic AESIs were identified. The statistically (although not clinically) significant decrease in axillary temperature and the relative reduction in other solicited symptoms following each round of the campaign compared with baseline is likely to reflect changes in environmental temperature and the seasonality of common childhood illnesses in The Gambia. The mean environmental temperature in October (>28°C) when the baseline data were collected, is higher than at the time of the two rounds of the campaign.^21^ Gastrointestinal, respiratory, and other common childhood illnesses (and malaria and malnutrition) peak towards the end of the wet season in September and October before falling in November and December.^22^ The increase in mild illnesses following the second round of the campaign compared with the first round probably reflects the additional reminders given to parents before the second round with the aim of maximising safety data completeness.

The type 2 poliovirus seroconversion rates of 70% following one dose and 91% following two doses of nOPV2 resulted in the seroprotection rate increasing to 97% over the two rounds of the campaign. A review of the results of studies of mOPV2 conducted between the 1950s and 1980s reported single-dose type 2 seroconversion rates of between 77% and 100% according to population group.^23^ Studies conducted in temperate climates tended to report higher seroconversion rates than those conducted in non-temperate regions, such as The Gambia, although differences in vaccine formulations and administration schedules limit the application of these data to the current study. A trial conducted in children aged 11–18 months in Mozambique reported a type 2 seroconversion rate of 68·0% (95% CI 56·8–77·5; 51 of 75 children) following a single dose of mOPV2.^24^ In IPV-primed Lithuanian children aged 1–5 years, a single-dose mOPV2 immune response rate of 71% (95% CI 54·5–83·9; 29 of 41 children) was reported.^25^ Finally, in infants aged 18–22 weeks in Panama, the single-dose immune response rate, combining infants who seroconverted and those who had a 4-fold rise in antibody titres was 92% (95% CI 84–96; 88 of 96 infants), and the rate after two doses was 97% (87–100; 38 of 39).^9^ Thus, the seroconversion and immune response rates following nOPV2 administration in this campaign were similar to those generated in trials of mOPV2.

Considering existing data on nOPV2, in the phase 2 trials in Panama from 2018 to 2019, 4 weeks after a first dose of nOPV2, 16 (94%, 95% CI 71–100) of 17 tOPV-primed and IPV-primed children had a serotype 2 immune response, and all 17 children (100%, 81–100) had a response following the second dose.^9^ In bOPV-primed and IPV-primed infants aged 18–22 weeks, 86% (95% CI 78–91; 108 of 126) and 98% (88–100; 44 of 45) had an immune response following one and two doses of nOPV2, respectively. The seroconversion rates in our study were similar to those reported from a 2021 population-based study in Tajikistan, which has a temperate climate, and to the rates reported from the clinical trial data in Panama.^8^, ^9^, ^26^ However, the 97% post-campaign seropositivity reported in this Article was much higher than the 38% (95% CI 34–43; 167 of 436) reported from a 2023 study in Liberia.^27^ Given the close geographical location of The Gambia and Liberia, and their similar socioeconomic statuses, these data are reassuring as they largely exclude lower vaccine immunogenicity as the primary driver in this setting. Technical issues related to the campaign or study in Liberia and inconsistent parental recall of the number of doses of nOPV2 children had received are alternative explanations.

Despite these immunogenicity data, a retrospective case-control study from Nigeria with data collected between 2017 and 2022 reported a one-dose effectiveness against acute flaccid paralysis of 12% (95% CI –2 to 25) for nOPV2 and 17% (3 to 29) for mOPV2.^28^ Data from the same study, and additional modelling of outbreaks, emphasise that increases in the number of high-quality supplementary immunisation activities remain crucial if large outbreaks in areas with low population immunity and high transmission rates are to be averted.^29^ Over the two rounds of the nOPV2 campaign, a third of children had an immune response to poliovirus serotypes 1 and 3. None of the children included in the analysis received bOPV over the study period, although passive exposure in the community to the viruses present in bOPV remains a possibility.

Nonetheless, in contrast to the homotypic type 2 response, the rates of heterotypic response were higher in children who were seropositive for the given poliovirus types at baseline, suggesting any cross-reactivity between the three poliovirus types tends to boost most effectively rather than prime (as has previously been reported).^30^ Less than 1% of children had type 2 poliovirus detected in their stool before the campaign—an indication of the level of exposure to the vaccine-derived poliovirus 2 detected through environmental surveillance in The Gambia. Less than half of children were excreting nOPV2 on day 7 following round 1 of the campaign and less than a fifth by day 28. These percentages are considerably lower than the 84·7% and 53·0% of infants excreting nOPV2 at the same timepoints in the phase 2 study of nOPV2 conducted in Panama.^9^ The differences might reflect differences in the age distribution of participants—younger children typically generate more robust mucosal immunity—or previous mucosal priming of the Gambian population with cVDPV2. Alternatively, inflammation associated with environmental enteropathy, measured with faecal and plasma biomarkers, has been shown to reduce poliovirus shedding following administration of an OPV.^31^ Differences in the prevalence of such enteric pathology, which is closely associated with socioeconomic status, between children in The Gambia and Panama is therefore an additional explanation. The percentage of children shedding nOPV2 was lower following the second round of the campaign, supporting the induction of mucosal immunity. In addition, the number of children excreting both the type 1 and the type 3 virus was reduced following both rounds of the campaign compared with the baseline. This might indicate a functional effect of the heterotypic responses or reflect the increasing interval since participants received their last dose of bOPV before enrolment.

Finally, the increased genetic stability of nOPV2 demonstrated in clinical trials^11^ is supported by data on nOPV2-associated cVDPV2 outbreaks under the emergency use listing.^32^ Although cVDPV2 associated with nOPV2 has been detected in Nigeria, the Democratic Republic of the Congo, and Burundi, the numbers of detections associated with acute flaccid paralysis or from environmental sampling are several-fold lower than would have been expected with mOPV2 given the number of doses delivered.^32^

This study has several strengths. It provides comprehensive real-world data on the use of nOPV2 in a campaign—complementing the clinical trial data—and has been submitted as part of the package to support the licensure and WHO prequalification of nOPV2. The conduct of the study in The Gambia, west Africa, and the inclusion of a broad cross-section of the target childhood population, including children with moderate and severe malnutrition, maximises the relevance of the findings to children in west Africa and other similar settings.

However, this study had some limitations. Although the number of children from whom data were collected was considerable, the expected frequency of both neurological and severe allergic AESIs, following administration of any OPV is low. Consequently, an absence of such events in this study should not be taken as excluding their future occurrence following the vaccination of large populations. Instead, ongoing data from reporting systems, strengthened in countries preparing for nOPV2 campaigns, will be required to provide frequency estimates for such rare events. In addition, we do not have a comprehensive description of the reasons why some children were not vaccinated during either round of the campaign, although we do not believe this loss is likely to substantially bias our findings.

In conclusion, when administered in the context of an outbreak response campaign in west Africa, nOPV2 was safe and well tolerated. It resulted in over 90% seroconversion over the two rounds of the campaign and generated intestinal mucosal immunity. These data support the licensure and WHO prequalification of this vaccine as the key tool in the global Polio Eradication Strategy.^3^

## Data sharing

The individual participant data that underlie the results reported in this Article, after de-identification (text, tables, figures, and appendices), will be shared. Individual participant data will be available from the time the Article is published and for 3 years subsequently. Supporting clinical documents, including the study protocol and the informed consent form, will be available. Researchers who provide a scientifically sound proposal will be allowed access to the individual participant data. Proposals should be directed to the corresponding author. These proposals will be reviewed and approved by a panel of senior scientific personnel from the Medical Research Council (MRC) Unit The Gambia at the London School of Hygiene & Tropical Medicine and by the Gambian Government and MRC Joint Ethics Committee. To gain access, data requesters will need to sign a data access agreement.

## Declaration of interests

We declare no competing interests.

## References

[1] WHO Global wild poliovirus 2016–2022. World Health Organization, Aug 30, 2022. https://polioeradication.org/wp-content/uploads/2022/09/weekly-polio-analyses-WPV-20220830.pdf.

[2] Hird TR, Grassly NC (2012). Systematic review of mucosal immunity induced by oral and inactivated poliovirus vaccines against virus shedding following oral poliovirus challenge. PLoS Pathog.

[3] WHO Global polio eradication initiative: strategy for the response to type 2 circulating vaccine-derived poliovirus 2020–2021. An addendum to the polio endgame strategy 2019. World Health Organization, 2023. http://polioeradication.org/wp-content/uploads/2020/04/Strategy-for-the-response-to-type-2-circulating-Vaccine-Derived-Poliovirus-20200406.pdf.

[4] Voorman A, Lyons H, Bennette C (2023). Analysis of population immunity to poliovirus following cessation of trivalent oral polio vaccine. Vaccine.

[5] Global Polio Eradication Initiative (2021). Global circulating vaccine-derived polioviruses. https://polioeradication.org/polio-today/.

[6] Macklin GR, O'Reilly KM, Grassly NC (2020). Evolving epidemiology of poliovirus serotype 2 following withdrawal of the serotype 2 oral poliovirus vaccine. Science.

[7] Yeh MT, Bujaki E, Dolan PT (2020). Engineering the live-attenuated polio vaccine to prevent reversion to virulence. Cell Host Microbe.

[8] De Coster I, Leroux-Roels I, Bandyopadhyay AS (2021). Safety and immunogenicity of two novel type 2 oral poliovirus vaccine candidates compared with a monovalent type 2 oral poliovirus vaccine in healthy adults: two clinical trials. Lancet.

[9] Sáez-Llorens X, Bandyopadhyay AS, Gast C (2021). Safety and immunogenicity of two novel type 2 oral poliovirus vaccine candidates compared with a monovalent type 2 oral poliovirus vaccine in children and infants: two clinical trials. Lancet.

[10] Van Damme P, De Coster I, Bandyopadhyay AS (2019). The safety and immunogenicity of two novel live attenuated monovalent (serotype 2) oral poliovirus vaccines in healthy adults: a double-blind, single-centre phase 1 study. Lancet.

[11] Wahid R, Mercer LD, De Leon T (2022). Genetic and phenotypic stability of poliovirus shed from infants who received novel type 2 or Sabin type 2 oral poliovirus vaccines in Panama: an analysis of two clinical trials. Lancet Microbe.

[12] WHO First ever vaccine listed under WHO emergency use. World Health Organization, Nov 13, 2020. https://www.who.int/news/item/13-11-2020-first-ever-vaccine-listed-under-who-emergency-use.

[13] Weldon WC, Oberste MS, Pallansch MA (2016). Standardized methods for detection of poliovirus antibodies. Methods Mol Biol.

[14] Harrington C, Sun H, Jeffries-Miles S (2021). Culture-independent detection of poliovirus in stool samples by direct RNA extraction. Microbiol Spectr.

[15] Division of AIDS, National Institute of Allergy and Infectious Diseases, National Institutes of Health, US Department of Health and Human Services (July, 2017). Division of AIDS (DAIDS) table for grading the severity of adult and pediatric adverse events. Corrected version 2.1. https://rsc.niaid.nih.gov/sites/default/files/daidsgradingcorrectedv21.pdf.

[16] Newcombe RG (1998). Two-sided confidence intervals for the single proportion: comparison of seven methods. Stat Med.

[17] Newcombe RG (1998). Interval estimation for the difference between independent proportions: comparison of eleven methods. Stat Med.

[18] Fagerland MW, Lydersen S, Laake P (2014). Recommended tests and confidence intervals for paired binomial proportions. Stat Med.

[19] Efron B (1987). Better bootstrap confidence intervals. J Am Stat Assoc.

[20] Pan African Clinical Trials Registry PACTR202010705577776: a phase 3, double-blind, randomized, controlled trial to assess the safety of a novel type 2 oral polio vaccine (nOPV2) in infants and young children and lot-to-lot consistency of the immune response to nOPV2 in infants in The Gambia. https://pactr.samrc.ac.za/TrialDisplay.aspx?TrialID=13419.

[21] Climate Change Knowledge Portal The Gambia. World Bank Group, 2022. https://climateknowledgeportal.worldbank.org/country/gambia.

[22] Brewster DR, Greenwood BM (1993). Seasonal variation of paediatric diseases in The Gambia, west Africa. Ann Trop Paediatr.

[23] Cáceres VM, Sutter RW (2001). Sabin monovalent oral polio vaccines: review of past experiences and their potential use after polio eradication. Clin Infect Dis.

[24] de Deus N, Capitine IPU, Bauhofer AFL (2022). Immunogenicity of reduced-dose monovalent type 2 oral poliovirus vaccine in Mocuba, Mozambique. J Infect Dis.

[25] Bandyopadhyay AS, Gast C, Brickley EB (2021). A randomized phase 4 study of immunogenicity and safety after monovalent oral type 2 sabin poliovirus vaccine challenge in children vaccinated with inactivated poliovirus vaccine in Lithuania. J Infect Dis.

[26] Mirzoev A, Macklin GR, Zhang Y (2022). Assessment of serological responses following vaccination campaigns with type 2 novel oral polio vaccine: a population-based study in Tajikistan in 2021. Lancet Glob Health.

[27] Kennedy SB, Macklin GR, Mason Ross G (2023). Poliovirus antibodies following two rounds of campaigns with a type 2 novel oral poliovirus vaccine in Liberia: a clustered, population-based seroprevalence survey. Lancet Glob Health.

[28] Cooper LV, Erbeto TB, Danzomo AA (2024). Effectiveness of poliovirus vaccines against circulating vaccine-derived type 2 poliomyelitis in Nigeria between 2017 and 2022: a case-control study. Lancet Infect Dis.

[29] Thompson KM, Kalkowska DA, Badizadegan K (2023). Looking back at prospective modeling of outbreak response strategies for managing global type 2 oral poliovirus vaccine (OPV2) cessation. Front Public Health.

[30] Ashkenazi A, Melnick JL (1962). Heterotypic antibody response after feeding of monovalent attenuated live poliovaccine. N Engl J Med.

[31] Grassly NC, Praharaj I, Babji S (2016). The effect of azithromycin on the immunogenicity of oral poliovirus vaccine: a double-blind randomised placebo-controlled trial in seronegative Indian infants. Lancet Infect Dis.

[32] Global Polio Eradication Initiative (March 16, 2023). GPEI statement on cVDPV2 detections in Burundi and Democratic Republic of the Congo. https://polioeradication.org/news-post/gpei-statement-on-cvdpv2-detections-in-burundi-and-democratic-republic-of-the-congo/.

