# Supplementary material for: Tolerability, safety, and immunogenicity of the novel oral polio vaccine type 2 in children aged 6 weeks to 59 months in an outbreak response campaign in The Gambia: an observational cohort study
Source: Lancet Infect Dis. 2024 Apr;24(4):417–26. doi: 10.1016/S1473-3099(23)00631-X (PMC10954559; doi:10.1016/S1473-3099(23)00631-X)
Supplement: Supplementary appendix [file mmc1.pdf]

THE LANCET
Infectious Diseases

Supplementary appendix

This appendix formed part of the original submission and has been peer reviewed. We post it as supplied by the authors.

Supplement to: Bashorun AO, Kotei L, Jawla O, et al. Tolerability, safety, and immunogenicity of the novel oral polio vaccine type 2 in children aged 6 weeks to 59 months in an outbreak response campaign in The Gambia: an observational cohort study. *Lancet Infect Dis* 2024; published online Jan 15. https://doi.org/10.1016/S1473-3099(23)00631-X.

Appendix

Table of Contents

Appendix: Study schedule 1

Appendix: Routine poliovirus immunization schedule in The Gambia 1

Appendix: Grading of adverse events over the first week following immunization 2

Appendix: Definitions of adverse events of special interest following nOPV2 administration.[1] 3

Appendix: US Centers for Disease Control and Prevention. 6

Appendix: Precision around given safety event rates according to sample size 7

Appendix: Probability of a given safety event occurring according to the true event rate and sample size for safety 7

Appendix: Baseline and post-vaccination solicited events 8

Appendix: Solicited events according to day post nOPV2 administration – round 1 10

Appendix: Solicited events according to day post nOPV2 administration – round 2 12

Appendix: Differences from baseline in solicited adverse events post campaign round 1 and round 2 – overall and according to specified strata 14

Appendix: Unsolicited adverse events by MedDRA system, organ, class, and preferred term 24

Appendix: Unsolicited adverse events in the 28 days following a first and second nOPV2 dose 27

Appendix: Rates of common unsolicited adverse events post round 1 and 2 of the nOPV2 campaign by MedDRA SOC or Preferred Term 28

Appendix: Serious adverse event listing 29

Appendix: Serotype-specific polio neutralizing antibody titres reverse cumulative distribution curves. 30

Appendix: Serotype-specific poliovirus PCR positivity in stool prior to and on day 7 and 28 following round 1 and round 2 of the nOPV2 campaign 31

References 32

Appendix: Study schedule

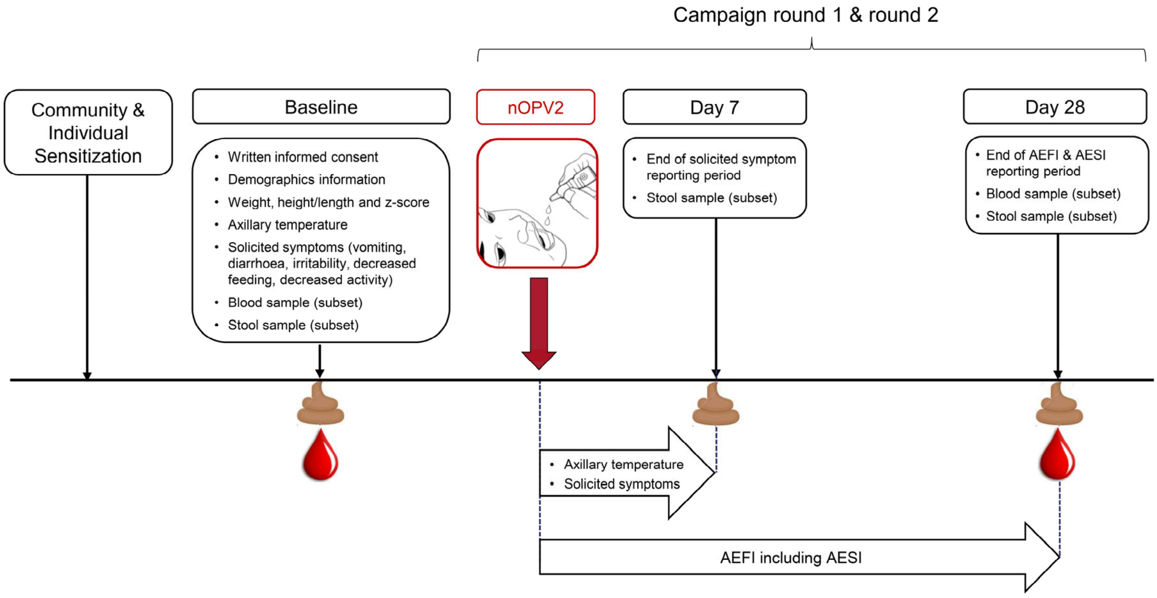

AEFI – adverse event following immunization; AESI – adverse event of special interest

Appendix: Routine poliovirus immunization schedule in The Gambia

Age	Vaccine
Birth	bOPV0
2 months	bOPV1
3 months	bOPV2
4 months	bOPV3<br>IPV1
9 months	bOPV4<br>IPV2
15 months	bOPV5

bOPV# - bivalent oral poliovirus vaccine dose number #; IPV# - inactivated poliovirus vaccine dose number #

Appendix: Grading of adverse events over the first week following immunization

Reactogenicity	Mild (Grade 1)	Moderate (Grade 2)	Severe (Grade 3)	Potentially Life Threatening (Grade 4)
Acute systemic allergic reaction‡	Localized urticaria (wheals) with no medical intervention indicated	Localized urticaria with medical intervention indicated OR Mild angioedema with no medical intervention indicated	Generalized urticaria OR Angioedema with medical intervention indicated OR Symptomatic mild bronchospasm	Acute anaphylaxis OR Life-threatening bronchospasm OR laryngeal edema
Axillary temperature	37.5 – 38.0°C	38.1 – 39.0°C	39.1 – 40.5°C	> 40.5°C
Vomiting	1 episode per 24 hours	2-5 episodes per 24 hours	≥6 episodes per 24 hours or requiring parenteral hydration	Life-threatening consequences (e.g., hypotensive shock)
Diarrhoea, <1 year of age	Liquid stools (more unformed than usual) but usual number of stools	Liquid stools with increased number of stools OR Mild dehydration	Liquid stools with moderate dehydration	Liquid stools resulting in severe dehydration with aggressive rehydration indicated OR Hypotensive shock
Diarrhoea, ≥1 year of age	Transient or intermittent episodes of unformed stools OR Increase of ≤ 3 stools over baseline per 24-hour period	Persistent episodes of unformed to watery stools OR Increase of 4 to 6 stools over baseline per 24-hour period	Increase of ≥ 7 stools per 24-hour period OR IV fluid replacement indicated	Life-threatening consequences (e.g., hypotensive shock)
Irritability	Crying more than usual but easily consoled	Crying more than usual and somewhat difficult to console	Continuous crying that is inconsolable	
Decreased activity	Slightly subdued, but responds normally to stimuli	Subdued and does not respond as readily as normal to stimuli	Lethargic	Obtunded
Decreased feeding	Eating less than normal	Missed 1 or 2 feeds/meals completely	Refuses ≥3 feeds/meals or refuses most feeds/meals	Life-threatening consequences OR Aggressive intervention indicated [e.g., tube feeding or total parenteral nutrition (TPN)]

Based on the U.S. Department of Health and Human Services, National Institutes of Health, National Institute of Allergy and Infectious Diseases, Division of AIDS. Division of AIDS (DAIDS) Table for Grading the Severity of Adult and Pediatric Adverse Events, Corrected Version 2.1. [July 2017]; ‡ Collected following vaccination only

Appendix: Definitions of adverse events of special interest following nOPV2 administration.[1]

AESI	
**Anaphylaxis**	Anaphylaxis is a severe allergic reaction that occurs within minutes to hours of vaccination. It is characterized by sudden onset of signs and symptoms with shock or collapse (altered consciousness, low blood pressure, weakness or absence of peripheral pulse, cold extremities). It may be accompanied with difficulty breathing, wheezing symptoms (noisy breathing), swelling (especially of the face, mouth, or throat), or skin rash (urticaria) that may be itchy. Patient may also have abdominal pain, vomiting or diarrhoea, and confusion.<br>Reporting criteria: Anaphylaxis diagnosed by a healthcare provider<br>*Sources:*<br>Gold MS, Gidudu J, Erlewyn-Lajeunesse M, Law B; Brighton Collaboration Working Group on Anaphylaxis. Can the Brighton Collaboration case definitions be used to improve the quality of Adverse Event Following Immunization (AEFI) reporting? Anaphylaxis as a case study. Vaccine. 2010 Jun 17;28(28):4487-98.<br>Rüggeberg JU, Gold MS, Bayas JM et al., Brighton Collaboration Anaphylaxis Working Group. Anaphylaxis: case definition and guidelines for data collection, analysis, and presentation of immunization safety data. Vaccine. 2007 Aug 1;25(31):5675-84.
**Acute Flaccid Paralysis**	AFP is a syndrome characterized by rapid onset of muscle weakness and limpness (flaccidity). This weakness can be found in one or more of an individual's extremities, or, more rarely, in the muscles of respiration and swallowing, progressing to maximum severity within 1-10 days. This includes Guillain-Barre Syndrome and transverse myelitis.<br>Reporting criteria: Any paralytic illness described by a healthcare provider<br>*Source:* World Health Organization. "WHO-recommended surveillance standard of poliomyelitis." http://www.who.int/immunization/monitoring_surveillance/burden/vpd/surveillance_type/active/poliomyelitis_standards/en/. Last accessed May 21, 2018.

**Aseptic meningitis**	Aseptic meningitis is the inflammation of the meninges, membranes covering the brain and spinal cord, in patients whose cerebral spinal fluid (CSF) test results are negative for routine bacterial cultures. Common symptoms include fever, vomiting, headaches, firm neck pain, sensitivity to light, and lethargy. Aseptic meningitis is generally a mild form of meningitis.<br>Reporting criteria: Aseptic meningitis as described by a healthcare provider<br>S*ources:*<br>BC case definition<br>Irani DN (August 2008). *"Aseptic meningitis and viral myelitis"*. Neurologic Clinics. 26 (3): 635–55, vii–viii. *doi*:*10.1016/j.ncl.2008.03.003*. *PMC 2728900*. *PMID 18657719*.<br>Norris CM, Danis PG, Gardner TD (May 1999). "Aseptic meningitis in the newborn and young infant". American Family Physician. 59 (10): 2761–70. PMID *10348069*.
**Encephalitis**	Encephalitis, or inflammation of the brain, is an acute onset of severe illness characterized by fever and altered mental status (encephalopathy). Focal neurological findings such as focal weakness, cranial nerve palsies, sensory deficits, or seizures can also be present. It occurs in approximately 2-30 days.<br>Reporting criteria: Encephalitis as described by a healthcare provider<br>Source: Brighton case definition
**Acute Disseminated Encephalomyelitis (ADEM)**	ADEM is a rapidly progressive neurological condition caused by a post-infectious inflammatory reaction in the brain and spinal cord. It is characterized by altered mental status and a decreased or complete loss of one or more cranial nerves, focal weakness and lack of muscle control or coordination of voluntary movements (ataxia). It occurs in approximately 2-30 days.<br>Reporting criteria: ADEM as described by a healthcare provider<br>Source: Brighton case definition

**Guillain-Barré syndrome**	Guillain-Barré syndrome (GBS) is a neurological condition in which a person's immune system attacks the peripheral nerves. It is characterized by ascending, flaccid (limp) weakness in legs to arms mostly in both sides of the body, may cause numbness (sensory loss) and eventually paralysis. It occurs in approximately 1-4 weeks<br><br>Reporting criteria: GBS as described by a healthcare provider<br><br>Source: Brighton case definition
**Myelitis/Transverse myelitis**	Myelitis/Transverse myelitis (TM) is a rare disorder caused by inflammation of the spinal cord. *Transverse* implies that the inflammation extends horizontally across the spinal cord. TM is characterized by: weakness in arms/legs; sensory symptoms such as numbness or tingling of the limbs, pain and discomfort as well as bowel or bladder dysfunction. The signs and symptoms depend on the area of spine involved and distribution of those symptoms may be symmetric or asymmetric affecting either legs, arms or both. It occurs within hours to weeks.<br><br>Reporting criteria: Myelitis/TM as described by a healthcare provider<br><br>Sources: Brighton case definition<br><br>West TW (October 2013). "Transverse myelitis--a review of the presentation, diagnosis, and initial management". Discovery Medicine. 16 (88): 167–77. PMID 24099672<br><br>https://www.hopkinsmedicine.org/neurology_neurosurgery/centers_clinics/transverse_myelitis/about-tm/what-is-transverse-myelitis.html
**Unexplained Death**	Death which remains unexplained after excluding other causes of death<br><br>Unexplained deaths including "Sudden Infant Death Syndrome" or SIDS in the first year of life, which remain unexplained after excluding other causes of death.<br><br>Source: Brighton Collaboration

Taken from The Global Polio Eradication Initiative: Guide for Surveillance of Adverse Events of Special Interest (AESI) during novel Oral Polio Vaccine type 2 (nOPV2) Use. October 27, 2020. https://polioeradication.org/wp-content/uploads/2022/06/nOPV2-AESI-surveillance.pdf

Appendix: US Centers for Disease Control and Prevention.

This activity was reviewed by CDC and was conducted consistent with applicable federal law and CDC policy (See e.g., 45 C.F.R. part 46, 21 C.F.R. part 56; 42 U.S.C. §241(d); 5 U.S.C. §552a; 44 U.S.C. §3501 et seq.).

Appendix: Precision around given safety event rates according to sample size

Event rate (%)	n = 1000	n = 5000	n = 10000
20.00	20.0 (17.6 - 22.6)	20.0 (18.9 - 21.1)	20.0 (19.2 - 20.8)
10.00	10.0 (8.3 - 12.0)	10.0 (9.2 - 10.9)	10.0 (9.4 - 10.6)
5.00	5.0 (3.8 - 6.5)	5.0 (4.4 - 5.6)	5.0 (4.6 - 5.4)
2.50	2.5 (1.7 - 3.7)	2.5 (2.1 - 3.0)	2.5 (2.2 - 2.8)
1.00	1.0 (0.5 - 1.8)	1.0 (0.8 - 1.3)	1.0 (0.8 - 1.2)
0.10	0.1 (0.02 - 0.5)	0.1 (0.04 - 0.2)	0.1 (0.05 - 0.2)
0.01	0.01 (0 - 0.4)	0.01 (0 - 0.1)	0.01 (0 - 0.1)

Appendix: Probability of a given safety event occurring according to the true event rate and sample size for safety

True event rate (% [frequency])		n = 1000		n = 5000		n = 10000	
		Probability of at least 1 event	Probability of at least 2 events	Probability of at least 1 event	Probability of at least 2 events	Probability of at least 1 event	Probability of at least 2 events
5	1 in 20	100.0	100.0	100.0	100.0	100.0	100.0
2	1 in 50	100.0	100.0	100.0	100.0	100.0	100.0
1	1 in 100	99.9	100.0	100.0	100.0	100.0	100.0
0.5	1 in 200	99.3	96.0	100.0	100.0	100.0	100.0
0.2	1 in 500	86.5	59.4	100.0	100.0	100.0	100.0
0.1	1 in 1000	63.2	26.4	99.3	96.0	100.0	100.0
0.05	1 in 2000	39.4	9.0	91.8	71.3	99.3	96.0
0.02	1 in 5000	18.1	1.8	63.2	26.4	86.5	59.4
0.01	1 in 10000	9.5	0.5	39.3	9.0	63.2	26.4
0.005	1 in 20000	4.9	0.1	22.1	2.6	39.3	9.0
0.002	1 in 50000	1.9	0.0	9.5	0.5	18.1	1.8
0.001	1 in 100000	1.0	0.0	4.9	0.1	9.5	0.4

Appendix: Baseline and post-vaccination solicited events

	Baseline n/N (%; 95% CI)	Post 1st dose n/N (%; 95% CI)	Post 2nd dose n/N (%; 95% CI)
**Axillary Temperature** (°C)			
Mean (95% CI)	36.38 (36.36 - 36.40)	36.27 (36.25 - 36.29)	36.22 (36.20 - 36.24)
< 37.5	5298/5498 (96.4%; 95.8 - 96.8)	4747/4843 (98.0%; 97.6 - 98.4)	4268/4346 (98.2%; 97.8 - 98.6)
37.5-38.0	120/5498 (2.2%; 1.8 - 2.6)	59/4843 (1.2%; 0.9 - 1.6)	57/4346 (1.3%; 1.0 - 1.7)
38.1-39.0	68/5498 (1.2%; 1.0 - 1.6)	26/4843 (0.5%; 0.4 - 0.8)	20/4346 (0.5%; 0.3 - 0.7)
39.1-40.5	12/5498 (0.2%; 0.1 - 0.4)	9/4843 (0.2%; 0.1 - 0.4)	1/4346 (<0.1%; 0.0 - 0.1)
> 40.5	0/5498 (0.0%; 0.0 - 0.1)	2/4843 (<0.1%; 0.0 - 0.2)	0/4346 (0.0%; 0.0 - 0.1)
**Immediate allergic reaction**			
No reaction		5034/5058 (99.5%; 99.3 - 99.7)	4882/4908 (99.5%; 99.2 - 99.6)
Grade 1		18/5058 (0.4%; 0.2 - 0.6)	21/4908 (0.4%; 0.3 - 0.7)
Grade 2		6/5058 (0.1%; 0.1 - 0.3)	5/4908 (0.1%; 0.0 - 0.2)
Grade 3		0/5058 (0.0%; 0.0 - 0.1)	0/4908 (0.0%; 0.0 - 0.1)
**Vomiting**			
No vomiting	5341/5504 (97.0%; 96.6 - 97.5)	5002/5059 (98.9%; 98.5 - 99.1)	4784/4909 (97.5%; 97.0 - 97.9)
Grade 1	122/5504 (2.2%; 1.9 - 2.6)	42/5059 (0.8%; 0.6 - 1.1)	80/4909 (1.6%; 1.3 - 2.0)
Grade 2	39/5504 (0.7%; 0.5 - 1.0)	15/5059 (0.3%; 0.2 - 0.5)	42/4909 (0.9%; 0.6 - 1.2)
Grade 3	2/5504 (<0.1%; 0.0 - 0.1)	0/5059 (0.0%; 0.0 - 0.1)	3/4909 (0.1%; 0.0 - 0.2)
**Diarrhoea**			
No diarrhoea	5108/5504 (92.8%; 92.1 - 93.5)	4863/5059 (96.1%; 95.6 - 96.6)	4679/4909 (95.3%; 94.7 - 95.9)
Grade 1	337/5504 (6.1%; 5.5 - 6.8)	146/5059 (2.9%; 2.5 - 3.4)	162/4909 (3.3%; 2.8 - 3.8)
Grade 2	57/5504 (1.0%; 0.8 - 1.3)	49/5059 (1.0%; 0.7 - 1.3)	67/4909 (1.4%; 1.1 - 1.7)
Grade 3	2/5504 (<0.1%; 0.0 - 0.1)	1/5059 (<0.1%; 0.0 - 0.1)	1/4909 (<0.1%; 0.0 - 0.1)
**Irritability**			
No irritability	5161/5504 (93.8%; 93.1 - 94.4)	5018/5059 (99.2%; 98.9 - 99.4)	4829/4909 (98.4%; 98.0 - 98.7)
Grade 1	312/5504 (5.7%; 5.1 - 6.3)	40/5059 (0.8%; 0.6 - 1.1)	74/4909 (1.5%; 1.2 - 1.9)
Grade 2	31/5504 (0.6%; 0.4 - 0.8)	1/5059 (<0.1%; 0.0 - 0.1)	6/4909 (0.1%; 0.1 - 0.3)
Grade 3	0/5504 (0.0%; 0.0 - 0.1)	0/5059 (0.0%; 0.0 - 0.1)	0/4909 (0.0%; 0.0 - 0.1)

	Baseline n/N (%; 95% CI)	Post 1st dose n/N (%; 95% CI)	Post 2nd dose n/N (%; 95% CI)
**Decreased feeding**			
Normal feeding	5031/5504 (91.4%; 90.6 - 92.1)	4960/5059 (98.0%; 97.6 - 98.4)	4769/4908 (97.2%; 96.7 - 97.6)
Grade 1	468/5504 (8.5%; 7.8 - 9.3)	99/5059 (2.0%; 1.6 - 2.4)	125/4908 (2.5%; 2.1 - 3.0)
Grade 2	5/5504 (0.1%; 0.0 - 0.2)	0/5059 (0.0%; 0.0 - 0.1)	14/4908 (0.3%; 0.2 - 0.5)
Grade 3	0/5504 (0.0%; 0.0 - 0.1)	0/5059 (0.0%; 0.0 - 0.1)	0/4908 (0.0%; 0.0 - 0.1)
**Decreased activity**			
Normal activity	5366/5504 (97.5%; 97.0 - 97.9)	5027/5059 (99.4%; 99.1 - 99.6)	4851/4909 (98.8%; 98.5 - 99.1)
Grade 1	137/5504 (2.5%; 2.1 - 2.9)	30/5059 (0.6%; 0.4 - 0.8)	54/4909 (1.1%; 0.8 - 1.4)
Grade 2	1/5504 (<0.1%; 0.0 - 0.1)	2/5059 (<0.1%; 0.0 - 0.1)	4/4909 (0.1%; 0.0 - 0.2)
Grade 3	0/5504 (0.0%; 0.0 - 0.1)	0/5059 (0.0%; 0.0 - 0.1)	0/4909 (0.0%; 0.0 - 0.1)

CI – confidence interval; °C – degrees Centigrade; n/N - number with the given grade of reaction/number assessed.

Appendix: Solicited events according to day post nOPV2 administration – round 1

	1-2 days<br>n/N (%; 95% CI)	3-5 days<br>n/N (%; 95% CI)	6-7 days<br>n/N (%; 95% CI)
**Axillary Temperature** (°C)			
Mean (95% CI)	36.31 (36.28 - 36.33)	36.26 (36.24 - 36.28)	36.22 (36.18 - 36.26)
< 37.5	1991/2036 (97.8%; 97.1 - 98.3)	1917/1943 (98.7%; 98.0 - 99.1)	360/367 (98.1%; 96.1 - 99.1)
37.5-38.0	31/2036 (1.5%; 1.1 - 2.2)	20/1943 (1.0%; 0.7 - 1.6)	6/367 (1.6%; 0.8 - 3.5)
38.1-39.0	14/2036 (0.7%; 0.4 - 1.2)	5/1943 (0.3%; 0.1 - 0.6)	1/367 (0.3%; 0.0 - 1.5)
39.1-40.5	0/2036 (0.0%; 0.0 - 0.2)	1/1943 (0.1%; 0.0 - 0.3)	0/367 (0.0%; 0.0 - 1.0)
> 40.5	0/2036 (0.0%; 0.0 - 0.2)	0/1943 (0.0%; 0.0 - 0.2)	0/367 (0.0%; 0.0 - 1.0)
**Vomiting**			
No vomiting	2150/2203 (97.6%; 96.9 - 98.2)	2085/2143 (97.3%; 96.5 - 97.9)	549/563 (97.5%; 95.9 - 98.5)
Grade 1	31/2203 (1.4%; 1.0 - 2.0)	42/2143 (2.0%; 1.5 - 2.6)	7/563 (1.2%; 0.6 - 2.5)
Grade 2	20/2203 (0.9%; 0.6 - 1.4)	16/2143 (0.7%; 0.5 - 1.2)	6/563 (1.1%; 0.5 - 2.3)
Grade 3	2/2203 (0.1%; 0.0 - 0.3)	0/2143 (0.0%; 0.0 - 0.2)	1/563 (0.2%; 0.0 - 1.0)
**Diarrhoea**			
No diarrhoea	2109/2203 (95.7%; 94.8 - 96.5)	2035/2143 (95.0%; 94.0 - 95.8)	535/563 (95.0%; 92.9 - 96.5)
Grade 1	68/2203 (3.1%; 2.4 - 3.9)	77/2143 (3.6%; 2.9 - 4.5)	17/563 (3.0%; 1.9 - 4.8)
Grade 2	26/2203 (1.2%; 0.8 - 1.7)	30/2143 (1.4%; 1.0 - 2.0)	11/563 (2.0%; 1.1 - 3.5)
Grade 3	0/2203 (0.0%; 0.0 - 0.2)	1/2143 (<0.1%; 0.0 - 0.3)	0/563 (0.0%; 0.0 - 0.7)
**Irritability**			
No irritability	2174/2203 (98.7%; 98.1 - 99.1)	2100/2143 (98.0%; 97.3 - 98.5)	555/563 (98.6%; 97.2 - 99.3)
Grade 1	26/2203 (1.2%; 0.8 - 1.7)	40/2143 (1.9%; 1.4 - 2.5)	8/563 (1.4%; 0.7 - 2.8)
Grade 2	3/2203 (0.1%; 0.0 - 0.4)	3/2143 (0.1%; 0.0 - 0.4)	0/563 (0.0%; 0.0 - 0.7)
Grade 3	0/2203 (0.0%; 0.0 - 0.2)	0/2143 (0.0%; 0.0 - 0.2)	0/563 (0.0%; 0.0 - 0.7)

	1-2 days<br>n/N (%; 95% CI)	3-5 days<br>n/N (%; 95% CI)	6-7 days<br>n/N (%; 95% CI)
**Decreased feeding**			
Normal feeding	2145/2203 (97.4%; 96.6 - 98.0)	2078/2142 (97.0%; 96.2 - 97.7)	546/563 (97.0%; 95.2 - 98.1)
Grade 1	50/2203 (2.3%; 1.7 - 3.0)	59/2142 (2.8%; 2.1 - 3.5)	16/563 (2.8%; 1.8 - 4.6)
Grade 2	8/2203 (0.4%; 0.2 - 0.7)	5/2142 (0.2%; 0.1 - 0.5)	1/563 (0.2%; 0.1 – 0.7)
Grade 3	0/2203 (0.0%; 0.0 - 0.2)	0/2142 (0.0%; 0.1 - 0.2)	0/563 (0.0%; 0.1 – 1.0)
**Decreased activity**			
Normal activity	2174/2203 (98.7%; 98.1 - 99.1)	2120/2143 (98.9%; 98.4 - 99.3)	557/563 (98.9%; 97.7 - 99.5)
Grade 1	26/2203 (1.2%; 0.8 - 1.7)	22/2143 (1.0%; 0.7 - 1.5)	6/563 (1.1%; 0.5 - 2.3)
Grade 2	3/2203 (0.1%; 0.0 - 0.4)	1/2143 (<0.1%; 0.0 - 0.3)	0/563 (0.0%; 0.0 - 0.7)
Grade 3	0/2203 (0.0%; 0.0 - 0.2)	0/2142 (0.0%; 0.0 - 0.2)	0/563 (0.0%; 0.0 - 0.7)

CI – confidence interval; °C – degrees Centigrade; n/N - number with the given grade of reaction/number assessed.

Appendix: Solicited events according to day post nOPV2 administration – round 2

	1-2 days<br>n/N (%; 95% CI)	3-5 days<br>n/N (%; 95% CI)	6-7 days<br>n/N (%; 95% CI)
**Axillary Temperature** (°C)			
Mean (95% CI)	36.25 (36.22 - 36.27)	36.19 (36.17 - 36.21)	36.23 (36.18 - 36.29)
< 37.5	1991/2036 (97.8%; 97.1 - 98.3)	1917/1943 (98.7%; 98.0 - 99.1)	360/367 (98.1%; 96.1 - 99.1)
37.5-38.0	31/2036 (1.5%; 1.1 - 2.2)	20/1943 (1.0%; 0.7 - 1.6)	6/367 (1.6%; 0.8 - 3.5)
38.1-39.0	14/2036 (0.7%; 0.4 - 1.2)	5/1943 (0.3%; 0.1 - 0.6)	1/367 (0.3%; 0.0 - 1.5)
39.1-40.5	0/2036 (0.0%; 0.0 - 0.2)	1/1943 (0.1%; 0.0 - 0.3)	0/367 (0.0%; 0.0 - 1.0)
> 40.5	0/2036 (0.0%; 0.0 - 0.2)	0/1943 (0.0%; 0.0 - 0.2)	0/367 (0.0%; 0.0 - 1.0)
**Vomiting**			
No vomiting	2150/2203 (97.6%; 96.9 - 98.2)	2085/2143 (97.3%; 96.5 - 97.9)	549/563 (97.5%; 95.9 - 98.5)
Grade 1	31/2203 (1.4%; 1.0 - 2.0)	42/2143 (2.0%; 1.5 - 2.6)	7/563 (1.2%; 0.6 - 2.5)
Grade 2	20/2203 (0.9%; 0.6 - 1.4)	16/2143 (0.7%; 0.5 - 1.2)	6/563 (1.1%; 0.5 - 2.3)
Grade 3	2/2203 (0.1%; 0.0 - 0.3)	0/2143 (0.0%; 0.0 - 0.2)	1/563 (0.2%; 0.0 - 1.0)
**Diarrhoea**			
No diarrhoea	2109/2203 (95.7%; 94.8 - 96.5)	2035/2143 (95.0%; 94.0 - 95.8)	535/563 (95.0%; 92.9 - 96.5)
Grade 1	68/2203 (3.1%; 2.4 - 3.9)	77/2143 (3.6%; 2.9 - 4.5)	17/563 (3.0%; 1.9 - 4.8)
Grade 2	26/2203 (1.2%; 0.8 - 1.7)	30/2143 (1.4%; 1.0 - 2.0)	11/563 (2.0%; 1.1 - 3.5)
Grade 3	0/2203 (0.0%; 0.0 - 0.2)	1/2143 (<0.1%; 0.0 - 0.3)	0/563 (0.0%; 0.0 - 0.7)
**Irritability**			
No irritability	2174/2203 (98.7%; 98.1 - 99.1)	2100/2143 (98.0%; 97.3 - 98.5)	555/563 (98.6%; 97.2 - 99.3)
Grade 1	26/2203 (1.2%; 0.8 - 1.7)	40/2143 (1.9%; 1.4 - 2.5)	8/563 (1.4%; 0.7 - 2.8)
Grade 2	3/2203 (0.1%; 0.0 - 0.4)	3/2143 (0.1%; 0.0 - 0.4)	0/563 (0.0%; 0.0 - 0.7)
Grade 3	0/2203 (0.0%; 0.0 - 0.2)	0/2143 (0.0%; 0.0 - 0.2)	0/563 (0.0%; 0.0 - 0.7)

	1-2 days<br>n/N (%; 95% CI)	3-5 days<br>n/N (%; 95% CI)	6-7 days<br>n/N (%; 95% CI)
**Decreased feeding**			
Normal feeding	2145/2203(97.4%; 96.6 - 98.0)	2078/2142(97.0%; 96.2 - 97.7)	546/563(97.0%; 95.2 - 98.1)
Grade 1	50/2203(2.3%; 1.7 - 3.0)	59/2142(2.8%; 2.1 - 3.5)	16/563(2.8%; 1.8 - 4.6)
Grade 2	8/2203(0.4%; 0.2 - 0.7)	5/2142(0.2%; 0.1 - 0.5)	1/563(0.2%; 0.0 - 1.0)
Grade 3	0/2203(0.0%; 0.0 - 0.2)	0/2142(0.0%; -0.0 - 0.2)	0/563(0.0%; 0.0 - 0.7)
**Decreased activity**			
Normal activity	2174/2203(98.7%; 98.1 - 99.1)	2120/2143(98.9%; 98.4 - 99.3)	557/563(98.9%; 97.7 - 99.5)
Grade 1	26/2203(1.2%; 0.8 - 1.7)	22/2143(1.0%; 0.7 - 1.5)	6/563(1.1%; 0.5 - 2.3)
Grade 2	3/2203(0.1%; 0.0 - 0.4)	1/2143(<0.1%; 0.0 - 0.3)	0/563(0.0%; 0.0 - 0.7)
Grade 3	0/2203(0.0%; 0.0 - 0.2)	0/2143(0.0%; 0.0 - 0.2)	0/563(0.0%; 0.0 - 0.7)

CI – confidence interval; °C – degrees Centigrade; n/N - number with the given grade of reaction/number assessed.

Appendix: Differences from baseline in solicited adverse events post campaign round 1 and round 2 – overall and according to specified strata

Point estimate for difference (Post-nOPV2 – baseline)	95% CI	Campaign round compared	Strata analysed	Number included in strata	Baseline	Post campaign round
**Axillary temperature (°C)§**						
-0.111	-0.155 to -0.067 ‖	Post 1 - Baseline	All participant	3838	36.39	36.28
-0.163	-0.208 to -0.119 ‖	Post 2 - Baseline	All participant	3838	36.39	36.22
-0.012	-0.083 to 0.059	Post 1 - Baseline	1-2 days post nOPV2 dose	1415	36.32	36.31
-0.147	-0.213 to -0.082 ‖	Post 2 - Baseline	1-2 days post nOPV2 dose	1797	36.40	36.25
-0.147	-0.211 to -0.082 ‖	Post 1 - Baseline	3-5 days post nOPV2 dose	1747	36.41	36.27
-0.17	-0.236 to -0.104 ‖	Post 2 - Baseline	3-5 days post nOPV2 dose	1746	36.36	36.19
-0.223	-0.333 to -0.113 ‖	Post 1 - Baseline	6-7 days post nOPV2 dose	675	36.44	36.22
-0.222	-0.377 to -0.066 ‖	Post 2 - Baseline	6-7 days post nOPV2 dose	294	36.46	36.23
-0.108	-0.169 to -0.046 ‖	Post 1 - Baseline	Male	1923	36.41	36.30
-0.168	-0.232 to-0.104 ‖	Post 2 - Baseline	Male	1923	36.41	36.24
-0.114	-0.177 to-0.051 ‖	Post 1 - Baseline	Female	1915	36.36	36.25
-0.158	-0.22 to-0.096 ‖	Post 2 - Baseline	Female	1915	36.36	36.21
-0.071	-0.181 to 0.038	Post 1 - Baseline	< 1-year-of-age	648	36.39	36.32
-0.07	-0.224 to 0.084	Post 2 - Baseline	< 1-year-of-age	346	36.36	36.29
-0.108	-0.176 to -0.039 ‖	Post 1 - Baseline	1-2-years-of-age	1828	36.37	36.27
-0.182	-0.251 to -0.114 ‖	Post 2 - Baseline	1-2-years-of-age	1815	36.39	36.21
-0.134	-0.199 to -0.069 ‖	Post 1 - Baseline	≥ 3-years-of-age and above	1362	36.40	36.27
-0.162	-0.224 to -0.099 ‖	Post 2 - Baseline	≥ 3-years-of-age and above	1677	36.39	36.23
-0.153	-0.288 to -0.019 ‖	Post 1 - Baseline	Severe malnutrition (z <-3 SD)¶	461	36.46	36.31
-0.21	-0.348 to -0.071 ‖	Post 2 - Baseline	Severe malnutrition (z <-3 SD)	461	36.46	36.25
-0.104	-0.161 to -0.048 ‖	Post 1 - Baseline	Moderate malnutrition (-2 ≤ z <-3 SD)	2270	36.38	36.27
-0.156	-0.213 to -0.099 ‖	Post 2 - Baseline	Moderate malnutrition (-2 ≤ z <-3 SD)	2270	36.38	36.22
-0.113	-0.208 to -0.018 ‖	Post 1 - Baseline	Normal nutrition (z >-2 SD)	810	36.35	36.24
-0.144	-0.24 to -0.049 ‖	Post 2 - Baseline	Normal nutrition (z >-2 SD)	810	36.35	36.21
-0.034	-0.3 to 0.232	Post 1 - Baseline	Previous bOPV doses: 0	89	36.40	36.36

Point estimate for difference (Post-nOPV2 – baseline)	95% CI		Campaign round compared	Strata analysed	Number included in strata	Baseline	Post campaign round
-0.063	-0.374 to 0.248		Post 2 - Baseline	Previous bOPV doses: 0	89	36.40	36.34
-0.064	-0.169 to 0.041		Post 1 - Baseline	Previous bOPV doses: 1-3	595	36.35	36.29
-0.113	-0.231 to 0.004		Post 2 - Baseline	Previous bOPV doses: 1-3	595	36.35	36.24
-0.122	-0.171 to -0.073	‖	Post 1 - Baseline	Previous bOPV doses: >3	3154	36.39	36.27
-0.175	-0.224 to -0.127	‖	Post 2 - Baseline	Previous bOPV doses: >3	3154	36.39	36.22
-0.171	-0.237 to -0.105	‖	Post 1 - Baseline	Ethnicity: Mandinka	1690	36.41	36.24
-0.209	-0.276 to -0.141	‖	Post 2 - Baseline	Ethnicity: Mandinka	1690	36.41	36.20
-0.064	-0.123 to -0.005	‖	Post 1 - Baseline	Ethnicity: Other	2148	36.37	36.30
-0.128	-0.187 to -0.068	‖	Post 2 - Baseline	Ethnicity: Other	2148	36.37	36.24
**Axillary Temperature > 37.5°C‡**							
-1.25%	-1.92 to -0.58%	‖	Post 1 - Baseline	All participant	3838	2.97%	1.72%
-1.46%	-2.13 to -0.79%	‖	Post 2 - Baseline	All participant	3838	2.97%	1.51%
0.35%	-0.69 to 1.40%		Post 1 - Baseline	1-2 days post nOPV2 dose	1415	1.98%	2.33%
-0.50%	-1.44 to 0.44%		Post 2 - Baseline	1-2 days post nOPV2 dose	1797	2.34%	1.84%
-1.77%	-2.80 to -0.74%	‖	Post 1 - Baseline	3-5 days post nOPV2 dose	1747	3.26%	1.49%
-2.18%	-3.20 to -1.15%	‖	Post 2 - Baseline	3-5 days post nOPV2 dose	1746	3.44%	1.26%
-3.26%	-4.97 to -1.53%	‖	Post 1 - Baseline	6-7 days post nOPV2 dose	675	4.30%	1.04%
-3.06%	-5.75 to -0.33%	‖	Post 2 - Baseline	6-7 days post nOPV2 dose	294	4.08%	1.02%
-0.73%	-1.69 to 0.24%		Post 1 - Baseline	Male	1923	2.76%	2.03%
-1.14%	-2.09 to -0.20%	‖	Post 2 - Baseline	Male	1923	2.76%	1.61%
-1.78%	-2.72 to -0.83%	‖	Post 1 - Baseline	Female	1915	3.19%	1.41%
-1.78%	-2.73 to -0.82%	‖	Post 2 - Baseline	Female	1915	3.19%	1.41%
-1.24%	-3.04 to 0.58%		Post 1 - Baseline	< 1-year-of-age	648	3.24%	2.01%
1.16%	-1.49 to 3.79%		Post 2 - Baseline	< 1-year-of-age	346	2.31%	3.47%
-2.02%	-3.09 to -0.95%	‖	Post 1 - Baseline	1-2-years-of-age	1828	3.83%	1.81%
-2.81%	-3.87 to -1.74%	‖	Post 2 - Baseline	1-2-years-of-age	1815	4.08%	1.27%
-0.22%	-1.14 to 0.70%		Post 1 - Baseline	≥ 3-years-of-age and above	1362	1.69%	1.47%

Point estimate for difference (Post-nOPV2 – baseline)	95% CI		Campaign round compared	Strata analysed	Number included in strata	Baseline	Post campaign round
-0.54%	-1.40 to 0.33%		Post 2 - Baseline	≥ 3-years-of-age and above	1677	1.91%	1.37%
-2.82%	-4.91 to 0.71%		Post 1 - Baseline	Severe malnutrition (z <-3 SD) ¶	461	3.91%	1.09%
-2.17%	-4.39 to 0.07%		Post 2 - Baseline	Severe malnutrition (z <-3 SD)	461	3.91%	1.74%
-1.41%	-2.26 to -0.56%	‖	Post 1 - Baseline	Moderate malnutrition (-2 ≤ z <-3 SD)	2270	2.95%	1.54%
-1.67%	-2.52 to -0.83%	‖	Post 2 - Baseline	Moderate malnutrition (-2 ≤ z <-3 SD)	2270	2.95%	1.28%
0%	-1.28 to 1.28%		Post 1 - Baseline	Normal nutrition (z >-2 SD)	810	1.73%	1.73%
0.25%	-1.12 to 1.61%		Post 2 - Baseline	Normal nutrition (z >-2 SD)	810	1.73%	1.98%
1.12%	-3.71 to 5.91%		Post 1 - Baseline	Previous bOPV doses: 0	89	1.12%	2.25%
3.37%	-2.36 to 8.96%		Post 2 - Baseline	Previous bOPV doses: 0	89	1.12%	4.49%
-1.68%	-3.34 to -0.01%	‖	Post 1 - Baseline	Previous bOPV doses: 1-3	595	3.03%	1.35%
-1.18%	-3.00 to 0.65%		Post 2 - Baseline	Previous bOPV doses: 1-3	595	3.03%	1.85%
-1.24%	-1.99 to -0.48%	‖	Post 1 - Baseline	Previous bOPV doses: >3	3154	3.01%	1.78%
-1.65%	-2.38 to -0.92%	‖	Post 2 - Baseline	Previous bOPV doses: >3	3154	3.01%	1.36%
-2.37%	-3.41 to -1.32%	‖	Post 1 - Baseline	Ethnicity: Mandinka	1690	3.55%	1.18%
-2.07%	-3.15 to -0.99%	‖	Post 2 - Baseline	Ethnicity: Mandinka	1690	3.55%	1.48%
-0.37%	-1.26 to 0.51%		Post 1 - Baseline	Ethnicity: Other	2148	2.51%	2.14%
-0.98%	-1.83 to -0.13%	‖	Post 2 - Baseline	Ethnicity: Other	2148	2.51%	1.54%
**Vomiting‡**							
-1.86%	-2.45 to -1.26%	‖	Post 1 - Baseline	All participant	4464	3.02%	1.17%
-0.40%	-1.09 to 0.29%		Post 2 - Baseline	All participant	4464	3.02%	2.62%
-1.20%	-2.03 to -0.37%	‖	Post 1 - Baseline	1-2 days post nOPV2 dose	1581	2.02%	0.82%
0.35%	-0.61 to 1.30%		Post 2 - Baseline	1-2 days post nOPV2 dose	1999	2.25%	2.60%
-1.02%	-1.78 to -0.27%	‖	Post 1 - Baseline	3-5 days post nOPV2 dose	2050	2.05%	1.02%
-0.91%	-2 to 0.18%		Post 2 - Baseline	3-5 days post nOPV2 dose	1971	3.55%	2.64%
-5.17%	-7.24 to -3.07%	‖	Post 1 - Baseline	6-7 days post nOPV2 dose	832	7.33%	2.16%
-1.42%	-3.75 to 0.93%		Post 2 - Baseline	6-7 days post nOPV2 dose	493	4.06%	2.64%
-1.65%	-2.53 to -0.78%	‖	Post 1 - Baseline	Male	2237	3.13%	1.48%

**Point estimate for difference** (Post-nOPV2 – baseline)	**95% CI**		**Campaign round compared**	**Strata analysed**	**Number included in strata**	**Baseline**	**Post campaign round**
-0.49%	-1.48 to 0.50%		Post 2 - Baseline	Male	2237	3.13%	2.64%
-2.07%	-2.88 to -1.25%	‖	Post 1 - Baseline	Female	2227	2.92%	0.85%
-0.31%	-1.28 to 0.65%		Post 2 - Baseline	Female	2227	2.92%	2.60%
-4.34%	-6.13 to -2.52%	‖	Post 1 - Baseline	< 1-year-of-age	761	5.52%	1.18%
-1.24%	-4.08 to 1.62%		Post 2 - Baseline	< 1-year-of-age	405	4.94%	3.70%
-1.91%	-2.79 to -1.03%	‖	Post 1 - Baseline	1-2-years-of-age	2145	3.12%	1.21%
0.14%	-0.98 to 1.26%		Post 2 - Baseline	1-2-years-of-age	2131	3.52%	3.66%
-0.58%	-1.42 to 0.27%		Post 1 - Baseline	≥ 3-years-of-age and above	1558	1.67%	1.09%
-0.83%	-1.65 to -0.01%	‖	Post 2 - Baseline	≥ 3-years-of-age and above	1928	2.08%	1.25%
-2.63%	-4.61 to -0.62%	‖	Post 1 - Baseline	Severe malnutrition (z <-3 SD) ¶	533	4.13%	1.50%
-2.25%	-4.31 to -0.18%	‖	Post 2 - Baseline	Severe malnutrition (z <-3 SD)	533	4.13%	1.88%
-2.21%	-2.99 to -1.42%	‖	Post 1 - Baseline	Moderate malnutrition (-2 ≤ z <-3 SD)	2624	3.24%	1.03%
-0.38%	-1.32 to 0.56%		Post 2 - Baseline	Moderate malnutrition (-2 ≤ z <-3 SD)	2624	3.24%	2.86%
-0.10%	-1.26 to 1.05%		Post 1 - Baseline	Normal nutrition (z >-2 SD)	972	1.65%	1.54%
1.34%	-0.01 to 2.68%		Post 2 - Baseline	Normal nutrition (z >-2 SD)	972	1.65%	2.98%
-4.85%	-10.29 to 0.76%		Post 1 - Baseline	Previous bOPV doses: 0	103	5.83%	0.97%
-0.97%	-7.14 to 5.24%		Post 2 - Baseline	Previous bOPV doses: 0	103	5.83%	4.85%
-3.46%	-5.12 to -1.78%	‖	Post 1 - Baseline	Previous bOPV doses: 1-3	694	4.32%	0.87%
-0.87%	-2.89 to 1.17%		Post 2 - Baseline	Previous bOPV doses: 1-3	694	4.32%	3.46%
-1.47%	-2.11 to -0.83%	‖	Post 1 - Baseline	Previous bOPV doses: >3	3667	2.70%	1.23%
-0.30%	-1.03 to 0.43%		Post 2 - Baseline	Previous bOPV doses: >3	3667	2.70%	2.40%
-2.31%	-3.25 to -1.37%	‖	Post 1 - Baseline	Ethnicity: Mandinka	1988	3.47%	1.16%
-2.16%	-3.11 to -1.22%	‖	Post 2 - Baseline	Ethnicity: Mandinka	1988	3.47%	1.31%
-1.49%	-2.26 to -0.72%	‖	Post 1 - Baseline	Ethnicity: Other	2476	2.67%	1.17%
1.01%	0.03 to 1.99%	*	Post 2 - Baseline	Ethnicity: Other	2476	2.67%	3.68%
**Diarrhoea‡**							
-3.09%	-3.98 to -2.20%	‖	Post 1 - Baseline	All participant	4464	6.81%	3.72%

**Point estimate for difference** (Post-nOPV2 – baseline)	**95% CI**		**Campaign round compared**	**Strata analysed**	**Number included in strata**	**Baseline**	**Post campaign round**
-2.04%	-3.00 to -1.07%	‖	Post 2 - Baseline	All participant	4464	6.81%	4.77%
-3.54%	-4.96 to -2.12%	‖	Post 1 - Baseline	1-2 days post nOPV2 dose	1581	6.20%	2.66%
-3.00%	-4.47 to -1.53%	‖	Post 2 - Baseline	1-2 days post nOPV2 dose	1999	7.40%	4.40%
-2.88%	-4.22 to -1.53%	‖	Post 1 - Baseline	3-5 days post nOPV2 dose	2050	7.32%	4.44%
-0.81%	-2.23 to 0.61%		Post 2 - Baseline	3-5 days post nOPV2 dose	1971	5.89%	5.07%
-2.64%	-4.76 to -0.52%	‖	Post 1 - Baseline	6-7 days post nOPV2 dose	832	6.61%	3.97%
-3.04%	-6.01 to -0.05%	‖	Post 2 - Baseline	6-7 days post nOPV2 dose	493	8.11%	5.07%
-2.82%	-4.09 to -1.54%	‖	Post 1 - Baseline	Male	2237	6.71%	3.89%
-1.25%	-2.64 to 0.14%		Post 2 - Baseline	Male	2237	6.71%	5.45%
-3.37%	-4.61 to -2.12%	‖	Post 1 - Baseline	Female	2227	6.92%	3.55%
-2.83%	-4.17 to -1.48%	‖	Post 2 - Baseline	Female	2227	6.92%	4.09%
-4.86%	-7.25 to -2.45%	‖	Post 1 - Baseline	< 1-year-of-age	761	9.46%	4.60%
0.49%	-3.05 to 4.03%		Post 2 - Baseline	< 1-year-of-age	405	7.16%	7.65%
-3.96%	-5.40 to -2.52%	‖	Post 1 - Baseline	1-2-years-of-age	2145	8.44%	4.48%
-3.14%	-4.78 to -1.51%	‖	Post 2 - Baseline	1-2-years-of-age	2131	9.53%	6.38%
-1.03%	-2.13 to 0.08%		Post 1 - Baseline	≥ 3-years-of-age and above	1558	3.27%	2.25%
-1.35%	-2.44 to -0.26%	‖	Post 2 - Baseline	≥ 3-years-of-age and above	1928	3.73%	2.39%
-6.94%	-9.81 to -4.02%	‖	Post 1 - Baseline	Severe malnutrition (z <-3 SD)¶	533	10.32%	3.38%
-6.00%	-9.18 to -2.79%	‖	Post 2 - Baseline	Severe malnutrition (z <-3 SD)	533	10.32%	4.32%
-2.71%	-3.86 to -1.55%	‖	Post 1 - Baseline	Moderate malnutrition (-2 ≤ z <-3 SD)	2624	6.67%	3.96%
-1.94%	-3.19 to -0.70%	‖	Post 2 - Baseline	Moderate malnutrition (-2 ≤ z <-3 SD)	2624	6.67%	4.73%
-1.54%	-3.35 to 0.27%		Post 1 - Baseline	Normal nutrition (z >-2 SD)	972	5.14%	3.60%
0.31%	-1.67 to 2.29%		Post 2 - Baseline	Normal nutrition (z >-2 SD)	972	5.14%	5.45%
0.97%	-5.24 to 7.14%		Post 1 - Baseline	Previous bOPV doses: 0	103	3.88%	4.85%
3.88%	-2.62 to 10.24%		Post 2 - Baseline	Previous bOPV doses: 0	103	3.88%	7.77%
-4.18%	-6.49 to -1.85%	‖	Post 1 - Baseline	Previous bOPV doses: 1-3	694	8.07%	3.89%
-0.58%	-3.42 to 2.27%		Post 2 - Baseline	Previous bOPV doses: 1-3	694	8.07%	7.49%

Point estimate for difference (Post-nOPV2 – baseline)	95% CI		Campaign round compared	Strata analysed	Number included in strata	Baseline	Post campaign round
-3%	-3.98 to -2.02%	‖	Post 1 - Baseline	Previous bOPV doses: >3	3667	6.65%	3.65%
-2.48%	-3.51 to -1.45%	‖	Post 2 - Baseline	Previous bOPV doses: >3	3667	6.65%	4.17%
-4.43%	-5.79 to -3.05%	‖	Post 1 - Baseline	Ethnicity: Mandinka	1988	7.65%	3.22%
-4.68%	-6.06 to -3.29%	‖	Post 2 - Baseline	Ethnicity: Mandinka	1988	7.65%	2.97%
-2.02%	-3.18 to -0.85%	‖	Post 1 - Baseline	Ethnicity: Other	2476	6.14%	4.12%
0.08%	-1.26 to 1.42%		Post 2 - Baseline	Ethnicity: Other	2476	6.14%	6.22%
**Irritability‡**							
-5.74%	-6.48 to -4.98%	‖	Post 1 - Baseline	All participant	4464	6.45%	0.72%
-4.77%	-5.58 to -3.95%	‖	Post 2 - Baseline	All participant	4464	6.45%	1.68%
-3.80%	-4.92 to -2.66%	‖	Post 1 - Baseline	1-2 days post nOPV2 dose	1581	4.68%	0.89%
-4.30%	-5.46 to -3.14%	‖	Post 2 - Baseline	1-2 days post nOPV2 dose	1999	5.70%	1.40%
-5.32%	-6.36 to -4.27%	‖	Post 1 - Baseline	3-5 days post nOPV2 dose	2050	5.71%	0.39%
-4.82%	-6.08 to -3.55%	‖	Post 2 - Baseline	3-5 days post nOPV2 dose	1971	6.85%	2.03%
-10.46%	-12.69 to -8.18%	‖	Post 1 - Baseline	6-7 days post nOPV2 dose	832	11.66%	1.20%
-6.29%	-8.92 to -3.61%	‖	Post 2 - Baseline	6-7 days post nOPV2 dose	493	7.71%	1.42%
-5.95%	-7.01 to -4.87%	‖	Post 1 - Baseline	Male	2237	6.57%	0.63%
-4.65%	-5.83 to -3.46%	‖	Post 2 - Baseline	Male	2237	6.57%	1.92%
-5.52%	-6.58 to -4.46%	‖	Post 1 - Baseline	Female	2227	6.33%	0.81%
-4.89%	-6.02 to -3.77%	‖	Post 2 - Baseline	Female	2227	6.33%	1.44%
-6.83%	-8.85 to -4.79%	‖	Post 1 - Baseline	< 1-year-of-age	761	7.88%	1.05%
-3.21%	-5.86 to -0.53%	‖	Post 2 - Baseline	< 1-year-of-age	405	5.19%	1.98%
-6.81%	-8.00 to -5.60%	‖	Post 1 - Baseline	1-2-years-of-age	2145	7.83%	1.03%
-6.15%	-7.51 to -4.78%	‖	Post 2 - Baseline	1-2-years-of-age	2131	8.63%	2.49%
-3.72%	-4.71 to -2.73%	‖	Post 1 - Baseline	≥ 3-years-of-age and above	1558	3.85%	0.13%
-3.58%	-4.57 to -2.58%	‖	Post 2 - Baseline	≥ 3-years-of-age and above	1928	4.31%	0.73%
-6.57%	-8.88 to -4.21%	‖	Post 1 - Baseline	Severe malnutrition (z <-3 SD)¶	533	7.32%	0.75%
-5.82%	-8.31 to -3.28%	‖	Post 2 - Baseline	Severe malnutrition (z <-3 SD)	533	7.32%	1.50%

Point estimate for difference (Post-nOPV2 – baseline)	95% CI		Campaign round compared	Strata analysed	Number included in strata	Baseline	Post campaign round
-6.02%	-7.04 to -5.00%	‖	Post 1 - Baseline	Moderate malnutrition (-2 ≤ z <-3 SD)	2624	6.90%	0.88%
-4.92%	-6.02 to -3.80%	‖	Post 2 - Baseline	Moderate malnutrition (-2 ≤ z <-3 SD)	2624	6.90%	1.98%
-3.70%	-5.01 to -2.38%	‖	Post 1 - Baseline	Normal nutrition (z >-2 SD)	972	4.01%	0.31%
-2.57%	-4.02 to -1.11%	‖	Post 2 - Baseline	Normal nutrition (z >-2 SD)	972	4.01%	1.44%
-4.85%	-10.29 to 0.76%		Post 1 - Baseline	Previous bOPV doses: 0	103	5.83%	0.97%
-4.85%	-10.29 to 0.76%		Post 2 - Baseline	Previous bOPV doses: 0	103	5.83%	0.97%
-4.90%	-6.76 to -3.01%	‖	Post 1 - Baseline	Previous bOPV doses: 1-3	694	5.76%	0.87%
-3.46%	-5.50 to -1.40%	‖	Post 2 - Baseline	Previous bOPV doses: 1-3	694	5.76%	2.31%
-5.92%	-6.75 to -5.08%	‖	Post 1 - Baseline	Previous bOPV doses: >3	3667	6.60%	0.68%
-5.02%	-5.92 to -4.11%	‖	Post 2 - Baseline	Previous bOPV doses: >3	3667	6.60%	1.58%
-8.00%	-9.25 to -6.73%	‖	Post 1 - Baseline	Ethnicity: Mandinka	1988	8.55%	0.55%
-7.95%	-9.21 to -6.67%	‖	Post 2 - Baseline	Ethnicity: Mandinka	1988	8.55%	0.60%
-3.92%	-4.81 to -3.02%	‖	Post 1 - Baseline	Ethnicity: Other	2476	4.77%	0.85%
-2.22%	-3.27 to -1.17%	‖	Post 2 - Baseline	Ethnicity: Other	2476	4.77%	2.54%
**Decreased feeding‡**							
-6.70%	-7.58 to -5.81%	‖	Post 1 - Baseline	All participant	4464	8.60%	1.90%
-5.67%	-6.62 to -4.72%	‖	Post 2 - Baseline	All participant	4464	8.60%	2.94%
-4.74%	-6.06 to -3.42%	‖	Post 1 - Baseline	1-2 days post nOPV2 dose	1581	6.39%	1.65%
-5.50%	-6.94 to -4.05%	‖	Post 2 - Baseline	1-2 days post nOPV2 dose	1999	8.40%	2.90%
-7.02%	-8.40 to -5.63%	‖	Post 1 - Baseline	3-5 days post nOPV2 dose	2050	9.46%	2.44%
-5.53%	-6.91 to -4.14%	‖	Post 2 - Baseline	3-5 days post nOPV2 dose	1971	8.47%	2.94%
-9.62%	-11.80 to -7.38%	‖	Post 1 - Baseline	6-7 days post nOPV2 dose	832	10.70%	1.08%
-6.69%	-9.70 to -3.63%	‖	Post 2 - Baseline	6-7 days post nOPV2 dose	493	9.74%	3.04%
-6.84%	-8.09 to -5.58%	‖	Post 1 - Baseline	Male	2237	8.63%	1.79%
-5.41%	-6.77 to -4.04%	‖	Post 2 - Baseline	Male	2237	8.63%	3.22%
-6.56%	-7.81 to -5.29%	‖	Post 1 - Baseline	Female	2227	8.58%	2.02%
-5.93%	-7.24 to -4.60%	‖	Post 2 - Baseline	Female	2227	8.58%	2.65%

Point estimate for difference (Post-nOPV2 – baseline)	95% CI	Campaign round compared	Strata analysed	Number included in strata	Baseline	Post campaign round
-4.47%	-6.32 to -2.60% ‖	Post 1 - Baseline	< 1-year-of-age	761	6.31%	1.84%
0.74%	-1.67 to 3.14%	Post 2 - Baseline	< 1-year-of-age	405	2.72%	3.46%
-8.25%	-9.63 to -6.86% ‖	Post 1 - Baseline	1-2-years-of-age	2145	10.35%	2.10%
-7.18%	-8.72 to -5.63% ‖	Post 2 - Baseline	1-2-years-of-age	2131	10.89%	3.71%
-5.65%	-7.07 to -4.21% ‖	Post 1 - Baseline	≥ 3-years-of-age and above	1558	7.32%	1.67%
-5.34%	-6.64 to -4.04% ‖	Post 2 - Baseline	≥ 3-years-of-age and above	1928	7.31%	1.97%
-11.82%	-14.79 to -8.76% ‖	Post 1 - Baseline	Severe malnutrition (z <-3 SD)¶	533	13.32%	1.50%
-11.45%	-14.51 to -8.29% ‖	Post 2 - Baseline	Severe malnutrition (z <-3 SD)	533	13.32%	1.88%
-6.33%	-7.48 to -5.17% ‖	Post 1 - Baseline	Moderate malnutrition (-2 ≤ z <-3 SD)	2624	8.42%	2.10%
-5.18%	-6.43 to -3.93% ‖	Post 2 - Baseline	Moderate malnutrition (-2 ≤ z <-3 SD)	2624	8.42%	3.24%
-3.09%	-4.52 to -1.64% ‖	Post 1 - Baseline	Normal nutrition (z >-2 SD)	972	4.32%	1.24%
-1.54%	-3.23 to 0.15%	Post 2 - Baseline	Normal nutrition (z >-2 SD)	972	4.32%	2.78%
0%	-3.73 to 3.73%	Post 1 - Baseline	Previous bOPV doses: 0	103	0.97%	0.97%
2.91%	-2.05 to 7.77%	Post 2 - Baseline	Previous bOPV doses: 0	103	0.97%	3.88%
-3.89%	-5.91 to -1.85% ‖	Post 1 - Baseline	Previous bOPV doses: 1-3	694	6.05%	2.16%
-3.03%	-5.21 to -0.83% ‖	Post 2 - Baseline	Previous bOPV doses: 1-3	694	6.05%	3.03%
-7.42%	-8.42 to -6.41% ‖	Post 1 - Baseline	Previous bOPV doses: >3	3667	9.30%	1.88%
-6.41%	-7.48 to -5.33% ‖	Post 2 - Baseline	Previous bOPV doses: >3	3667	9.30%	2.89%
-8.55%	-9.99 to -7.10% ‖	Post 1 - Baseline	Ethnicity: Mandinka	1988	10.41%	1.86%
-8.70%	-10.13 to -7.25% ‖	Post 2 - Baseline	Ethnicity: Mandinka	1988	10.41%	1.71%
-5.21%	-6.31 to -4.10% ‖	Post 1 - Baseline	Ethnicity: Other	2476	7.15%	1.94%
-3.23%	-4.49 to -1.97% ‖	Post 2 - Baseline	Ethnicity: Other	2476	7.15%	3.92%
**Decreased activity‡**						
-1.88%	-2.40 to -1.36% ‖	Post 1 - Baseline	All participant	4464	2.51%	0.63%
-1.28%	-1.84 to -0.71% ‖	Post 2 - Baseline	All participant	4464	2.51%	1.23%
-0.89%	-1.56 to -0.21% ‖	Post 1 - Baseline	1-2 days post nOPV2 dose	1581	1.33%	0.44%
-0.65%	-1.49 to 0.19%	Post 2 - Baseline	1-2 days post nOPV2 dose	1999	2.10%	1.45%

Point estimate for difference (Post-nOPV2 – baseline)	95% CI		Campaign round compared	Strata analysed	Number included in strata	Baseline	Post campaign round
-2.24%	-3.10 to -1.38%	‖	Post 1 - Baseline	3-5 days post nOPV2 dose	2050	3.07%	0.83%
-1.73%	-2.59 to -0.86%	‖	Post 2 - Baseline	3-5 days post nOPV2 dose	1971	2.79%	1.07%
-2.89%	-4.23 to -1.52%	‖	Post 1 - Baseline	6-7 days post nOPV2 dose	832	3.37%	0.48%
-2.03%	-3.87 to -0.17%	‖	Post 2 - Baseline	6-7 days post nOPV2 dose	493	3.04%	1.01%
-1.65%	-2.37 to -0.94%	‖	Post 1 - Baseline	Male	2237	2.28%	0.63%
-0.85%	-1.66 to -0.04%	‖	Post 2 - Baseline	Male	2237	2.28%	1.43%
-2.11%	-2.88 to -1.34%	‖	Post 1 - Baseline	Female	2227	2.74%	0.63%
-1.71%	-2.51 to -0.90%	‖	Post 2 - Baseline	Female	2227	2.74%	1.03%
-0.92%	-1.98 to 0.14%		Post 1 - Baseline	< 1-year-of-age	761	1.45%	0.53%
0.74%	-0.54 to 2.01%		Post 2 - Baseline	< 1-year-of-age	405	0.25%	0.99%
-2.56%	-3.37 to -1.76%	‖	Post 1 - Baseline	1-2-years-of-age	2145	3.08%	0.51%
-1.27%	-2.22 to -0.32%	‖	Post 2 - Baseline	1-2-years-of-age	2131	3.14%	1.88%
-1.41%	-2.30 to -0.53%	‖	Post 1 - Baseline	≥ 3-years-of-age and above	1558	2.25%	0.83%
-1.71%	-2.47 to -0.95%	‖	Post 2 - Baseline	≥ 3-years-of-age and above	1928	2.28%	0.57%
-3.38%	-5.28 to -1.45%	‖	Post 1 - Baseline	Severe malnutrition (z <-3 SD)¶	533	4.13%	0.75%
-3.00%	-4.98 to -1%	‖	Post 2 - Baseline	Severe malnutrition (z <-3 SD)	533	4.13%	1.13%
-1.98%	-2.68 to -1.28%	‖	Post 1 - Baseline	Moderate malnutrition (-2 ≤ z <-3 SD)	2624	2.63%	0.65%
-1.41%	-2.16 to -0.66%	‖	Post 2 - Baseline	Moderate malnutrition (-2 ≤ z <-3 SD)	2624	2.63%	1.22%
-0.10%	-0.88 to 0.68%		Post 1 - Baseline	Normal nutrition (z >-2 SD)	972	0.72%	0.62%
0.72%	-0.25 to 1.68%		Post 2 - Baseline	Normal nutrition (z >-2 SD)	972	0.72%	1.44%
0%	-2.64 to 2.64%		Post 1 - Baseline	Previous bOPV doses: 0	103	0%	0%
0.97%	-2.28 to 4.18%		Post 2 - Baseline	Previous bOPV doses: 0	103	0%	0.97%
-0.72%	-1.81 to 0.37%		Post 1 - Baseline	Previous bOPV doses: 1-3	694	1.30%	0.58%
-0.29%	-1.41 to 0.84%		Post 2 - Baseline	Previous bOPV doses: 1-3	694	1.30%	1.01%
-2.15%	-2.76 to -1.55%	‖	Post 1 - Baseline	Previous bOPV doses: >3	3667	2.81%	0.65%
-1.53%	-2.18 to -0.87%	‖	Post 2 - Baseline	Previous bOPV doses: >3	3667	2.81%	1.28%
-3.17%	-4.06 to -2.27%	‖	Post 1 - Baseline	Ethnicity: Mandinka	1988	3.67%	0.50%

Point estimate for difference (Post-nOPV2 – baseline)	95% CI		Campaign round compared	Strata analysed	Number included in strata	Baseline	Post campaign round
-3.07%	-3.97 to -2.16%	‖	Post 2 - Baseline	Ethnicity: Mandinka	1988	3.67%	0.60%
-0.85%	-1.45 to -0.24%	‖	Post 1 - Baseline	Ethnicity: Other	2476	1.58%	0.73%
0.16%	-0.56 to 0.88%		Post 2 - Baseline	Ethnicity: Other	2476	1.58%	1.74%

CI – confidence interval; bOPV – bivalent oral poliovirus vaccine; SD – standard deviation; § confidence intervals calculated with the constant variance assumptions of a paired t-test; ‡ confidence intervals calculated with Wald Bonnet-Price for paired proportions.[2]; weight for height z-scores calculated based on WHO standards.[3]; ‖ lower temperature or frequency of event post-nOPV2 campaign than at baseline; * higher frequency of event post-nOPV2 campaign than at baseline.

Appendix: Unsolicited adverse events by MedDRA system, organ, class, and preferred term

	Post 1st dose<br>N=5061<br>n (%)	Post 2nd dose<br>N=4911<br>n (%)
**Any Adverse Events**		
Total	322 (6.4%)	624 (12.7%)
**Infections and infestations**		
Total	162 (3.2%)	422 (8.6%)
Upper respiratory tract infection	52 (1.0%)	147 (3.0%)
Febrile infection	26 (0.5%)	42 (0.9%)
Pneumonia	18 (0.4%)	39 (0.8%)
Gastroenteritis	17 (0.3%)	97 (2.0%)
Conjunctivitis	11 (0.2%)	18 (0.4%)
Malaria	10 (0.2%)	0
Abscess	8 (0.2%)	14 (0.3%)
Skin infection	8 (0.2%)	6 (0.1%)
Helminthic infection	6 (0.1%)	27 (0.5%)
Oral candidiasis	3 (0.1%)	17 (0.3%)
Acrodermatitis	2 (<0.1%)	4 (0.1%)
Otitis media acute	2 (<0.1%)	1 (<0.1%)
Cellulitis	1 (<0.1%)	1 (<0.1%)
Gingivitis	1 (<0.1%)	0
Impetigo	1 (<0.1%)	9 (0.2%)
Otitis media	1 (<0.1%)	0
Rash pustular	1 (<0.1%)	15 (0.3%)
Tonsillitis	1 (<0.1%)	3 (0.1%)
Varicella	1 (<0.1%)	8 (0.2%)
Abscess neck	0	2 (<0.1%)
Dysentery	0	1 (<0.1%)
Eczema infected	0	3 (0.1%)
Furuncle	0	8 (0.2%)
Mumps	0	1 (<0.1%)
Nasopharyngitis	0	1 (<0.1%)
Sepsis	0	1 (<0.1%)
Tinea infection	0	7 (0.1%)
Urinary tract infection	0	2 (<0.1%)
**Respiratory, thoracic, and mediastinal disorders**		
Total	89 (1.8%)	87 (1.8%)
Cough	88 (1.7%)	86 (1.8%)
Asthma	1 (<0.1%)	0
Oropharyngeal pain	0	1 (<0.1%)
**Gastrointestinal disorders**		
Total	73 (1.4%)	154 (3.1%)

Appendix: Unsolicited adverse events by MedDRA system, organ, class, and preferred term

	Post 1st dose N=5061 n (%)	Post 2nd dose N=4911 n (%)
Diarrhoea	50 (1.0%)	100 (2.0%)
Vomiting	16 (0.3%)	33 (0.7%)
Abdominal pain	3 (0.1%)	4 (0.1%)
Mouth ulceration	2 (<0.1%)	2 (<0.1%)
Abdominal distension	1 (<0.1%)	1 (<0.1%)
Diarrhoea haemorrhagic	1 (<0.1%)	6 (0.1%)
Dyspepsia	1 (<0.1%)	0
Angular cheilitis	0	2 (<0.1%)
Aphthous ulcer	0	2 (<0.1%)
Constipation	0	2 (<0.1%)
Oral pain	0	2 (<0.1%)
Stomatitis	0	1 (<0.1%)
Toothache	0	3 (0.1%)
**Skin and subcutaneous tissue disorders**		
Total	15 (0.3%)	21 (0.4%)
Skin disorder	9 (0.2%)	0
Dermatitis diaper	3 (0.1%)	4 (0.1%)
Urticaria	2 (<0.1%)	3 (0.1%)
Rash	1 (<0.1%)	1 (<0.1%)
Dermatitis atopic	0	3 (0.1%)
Dermatitis contact	0	3 (0.1%)
Rash maculo-papular	0	4 (0.1%)
Rash vesicular	0	3 (0.1%)
**General disorders and administration site conditions**		
Total	11 (0.2%)	23 (0.5%)
Pyrexia	11 (0.2%)	22 (0.4%)
Cheek swelling	0	1 (<0.1%)
**Injury, poisoning and procedural complications**		
Total	8 (0.2%)	9 (0.2%)
Injury	4 (0.1%)	4 (0.1%)
Arthropod bite	1 (<0.1%)	0
Bite	1 (<0.1%)	0
Foreign body in ear	1 (<0.1%)	0
Skin laceration	1 (<0.1%)	0
Accidental poisoning	0	1 (<0.1%)
Arthropod sting	0	1 (<0.1%)
Thermal burn	0	2 (<0.1%)
Wound	0	1 (<0.1%)
**Ear and labyrinth disorders**		

Appendix: Unsolicited adverse events by MedDRA system, organ, class, and preferred term

	Post 1st dose<br>N=5061<br>n (%)	Post 2nd dose<br>N=4911<br>n (%)
Total	4 (0.1%)	7 (0.1%)
Otorrhoea	2 (<0.1%)	4 (0.1%)
Ear disorder	1 (<0.1%)	0
Ear pain	1 (<0.1%)	3 (0.1%)
**Blood and lymphatic system disorders**		
Total	3 (0.1%)	3 (0.1%)
Anaemia	3 (0.1%)	3 (0.1%)
**Eye disorders**		
Total	2 (<0.1%)	0
Eye disorder	2 (<0.1%)	0
**Metabolism and nutrition disorders**		
Total	2 (<0.1%)	0
Decreased appetite	1 (<0.1%)	0
Feeding disorder	1 (<0.1%)	0
**Nervous system disorders**		
Total	2 (<0.1%)	1 (<0.1%)
Somnolence	2 (<0.1%)	0
Headache	0	1 (<0.1%)
**Metabolism and nutrition disorders**		
Total	0	3 (0.1%)
Decreased appetite	0	3 (0.1%)

Appendix: Unsolicited adverse events in the 28 days following a first and second nOPV2 dose

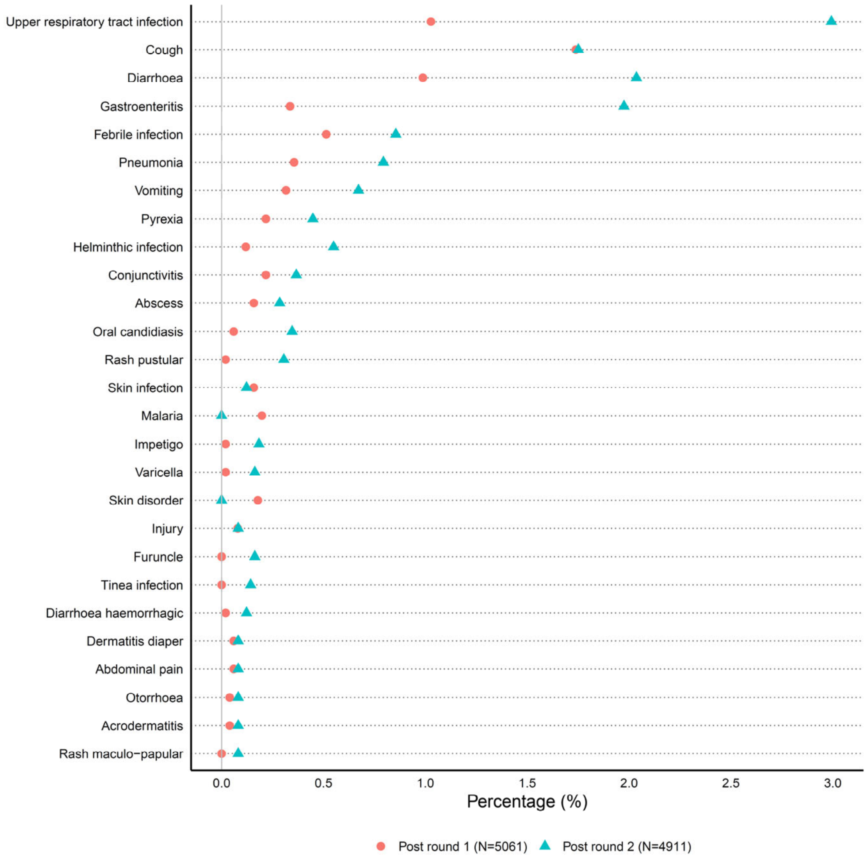

	Post round 1	Post round 2
Upper respiratory tract infection	52	147
Cough	88	86
Diarrhoea	50	100
Gastroenteritis	17	97
Febrile infection	26	42
Pneumonia	18	39
Vomiting	16	33
Pyrexia	11	22
Helminthic infection	6	27
Conjunctivitis	11	18
Abscess	8	14
Oral candidiasis	3	17
Rash pustular	1	15
Skin infection	8	6
Malaria	10	0
Impetigo	1	9
Varicella	1	8
Skin disorder	9	0
Injury	4	4
Furuncle	0	8
Tinea infection	0	7
Diarrhoea haemorrhagic	1	6
Dermatitis diaper	3	4
Abdominal pain	3	4
Otorrhoea	2	4
Acrodermatitis	2	4
Rash maculo−papular	0	4

Appendix: Rates of common unsolicited adverse events post round 1 and 2 of the nOPV2 campaign by MedDRA SOC or Preferred Term

		Post round 1		Post round 2	
	**Interval**	**Rate per 1,000**	**95% CI‡**	**Rate per 1,000**	**95% CI‡**
Infections and infestations	Unadjusted	33.59	28.45 to 38.93‖	98.76	89.19 to 108.53‖
	≤ 7 days	13.04	9.88 to 16.4‖	25.05	20.77 to 29.53‖
	8 to 14 days	7.11	4.74 to 9.68‖	16.09	12.42 to 20.16‖
	≥ 15 days	13.44	10.27 to 16.8‖	57.63	50.7 to 64.75‖
Gastrointestinal disorders	Unadjusted	14.62	11.26 to 17.98‖	32.58	27.49 to 37.87‖
	≤ 7 days	8.3	5.93 to 10.87‖	10.59	8.1 to 13.9
	8 to 14 days	2.77	1.6 to 4.6	5.91	4.1 to 8.5
	≥ 15 days	3.56	2.3 to 5.6	16.09	12.62 to 19.75‖
Respiratory, thoracic, and mediastinal disorders	Unadjusted	17.59	14.3 to 21.6	18.73	14.86 to 22.81‖
	≤ 7 days	8.5	6.3 to 11.4	7.33	5.3 to 10.1
	8 to 14 days	3.56	2.3 to 5.6	2.65	1.5 to 4.5
	≥ 15 days	5.53	3.8 to 8	8.76	6.5 to 11.8
Upper respiratory tract infection or cough	Unadjusted	27.66	23.12 to 32.4‖	49.89	43.58 to 56.61
	≤ 7 days	12.65	9.9 to 16.1	15.27	12.2 to 19.1
	8 to 14 days	4.54	2.77 to 6.72‖	7.74	5.6 to 10.6
	≥ 15 days	10.47	8 to 13.7	26.88	22.4 to 31.56‖
Gastroenteritis	Unadjusted	13.24	10.08 to 16.6‖	41.13	35.43 to 47.04‖
	≤ 7 days	7.11	4.74 to 9.68‖	15.27	11.81 to 18.94‖
	8 to 14 days	1.98	1.1 to 3.6	8.55	6.11 to 11.2‖
	≥ 15 days	4.15	2.7 to 6.3	17.31	13.64 to 21.18‖
Febrile infection	Unadjusted	7.31	5.3 to 10.1	13.24	10.18 to 16.49‖
	≤ 7 days	3.16	1.9 to 5.1	3.46	2.2 to 5.5
	8 to 14 days	1.38	0.7 to 2.9	1.83	1 to 3.5
	≥ 15 days	2.77	1.6 to 4.6	7.94	5.8 to 10.8

MedDRA – Medical Dictionary of Regulatory Activities; SOC – System, Organ, Class; CI – confidence interval; ‡ - Wilson score confidence interval used when participants had up to 1 event (unless otherwise indicated); ‖ - Bootstrap confidence intervals used when some participants had > 1 of a given event in the absence of a definitive theoretical distribution for the zero inflated counts. 100 000 samples were drawn for each event*interval combination. For each bootstrapped sample we log transformed the rate estimate (to allow for heteroscedastic rates), calculated the percentile bootstrap sample of the 100,000 samples and anti-logged to return to the correct scaling for a rate. The rates were large enough such that there were no bootstrapped zero rates.

Appendix: Serious adverse event listing

Event term	Round	Age (months)	Sex	Number of days post vaccination	Related to nOPV2 administration	Duration of event (days)	Admitted to hospital/clinic overnight	Outcome
Pneumonia	1	5	Female	6	No	10	Hospital/clinic admission	Recovered
Pneumonia	1	11	Male	3	No	8	Hospital/clinic admission	Recovered
Malaria	1	11	Male	3	No	8	Hospital/clinic admission	Recovered
Pneumonia	1	7	Male	19	No	7	Hospital/clinic admission	Recovered
Pneumonia	1	10	Female	17	No	7	Hospital/clinic admission	Recovered
Anaemia	1	10	Female	17	No	7	Hospital/clinic admission	Recovered
Abdominal pain	1	27	Female	8	No	2	Hospital/clinic admission	Recovered
Pneumonia	2	10	Female	29	No	2	Hospital/clinic admission	Recovered
Anaemia	2	5	Male	33	No	5	Hospital/clinic admission	Recovered
Pneumonia	2	5	Male	33	No	5	Hospital/clinic admission	Recovered
Pneumonia	2	5	Male	11	No	4	Hospital/clinic admission	Recovered
Anaemia	2	5	Male	11	No	4	Hospital/clinic admission	Recovered
Gastroenteritis‡	2	1	Female	3	No	1	Died	Died
Sepsis	2	1	Female	4	No	5	Hospital/clinic admission	Recovered
Pneumonia	2	4	Female	0	No	6	Hospital/clinic admission	Recovered

‡ This infant developed diarrhoea and vomiting following the second round of the campaign. The child had no symptoms following the first campaign round. The child was pronounced dead on arrival at a health facility. The symptoms and signs described were felt to be consistent with a diagnosis of infectious gastroenteritis. Given cultural practices and early burial no samples could be collected for analysis and a post-mortem was not performed to confirm the diagnosis.

Appendix: Serotype-specific polio neutralizing antibody titres reverse cumulative distribution curves.

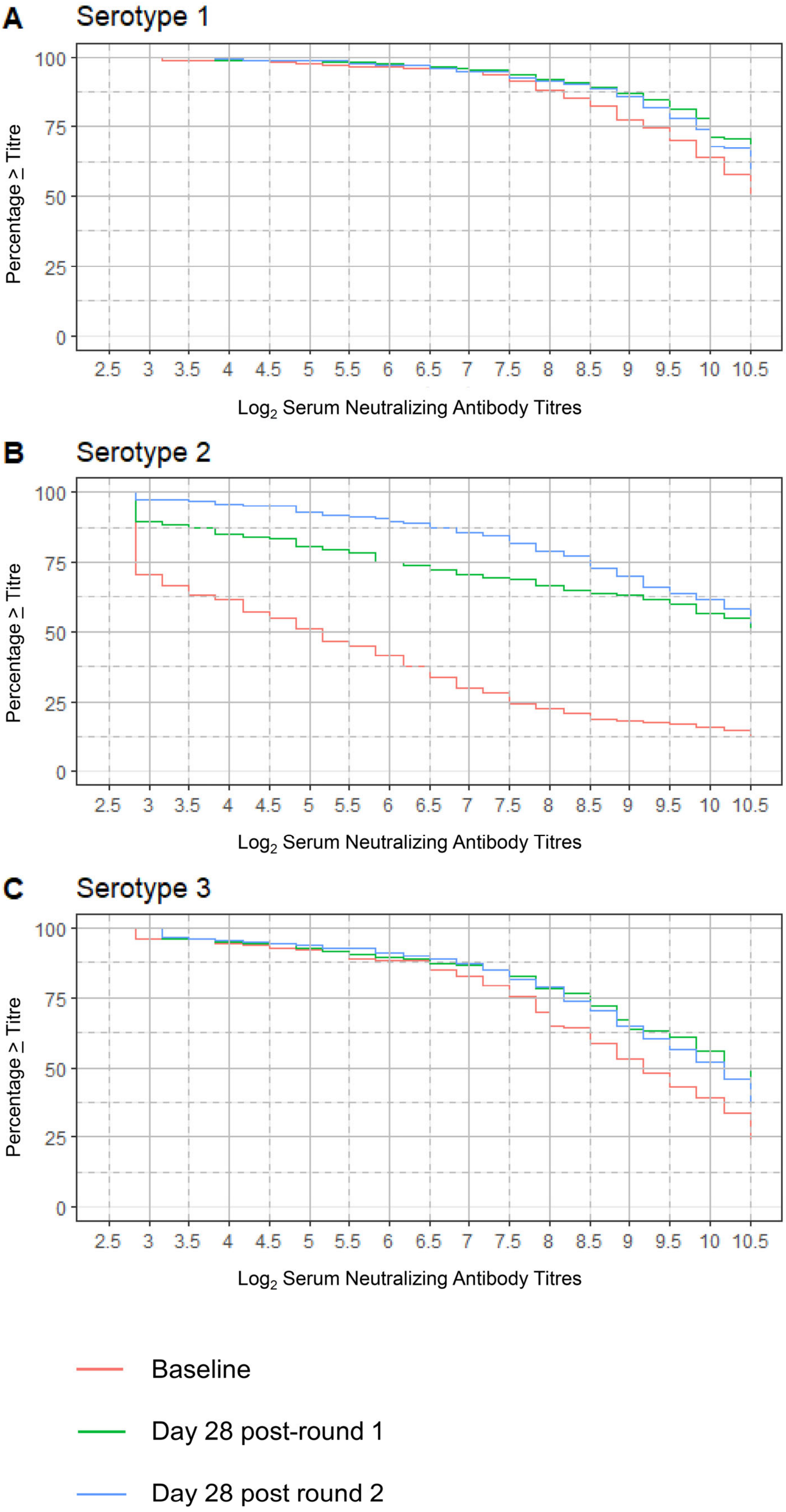

Appendix: Serotype-specific poliovirus PCR positivity in stool prior to and on day 7 and 28 following round 1 and round 2 of the nOPV2 campaign

	Day post nOPV2 campaign	Round 1 % (n/N) (95% CI)	Round 2 % (n/N) (95% CI)	Difference (post-round 1 – post-round 2) % (95% CI)
**Serotype 1**	**Baseline**	1.3% (15/1162) (0.8 to 2.1)	-	-
	**Day 7**	0.8% (5/658) (0.3 to 1.8)	1.1% (5/459) (0.5 to 2.5)	0.3% (-0.8 to 1.7)
	**Day 28**	0.3% (1/376) (0.05 to 1.5)	0.7% (2/272) (0.2 to 2.6)	0.5% (-0.8 to 2.2)
**Serotype 2**	**Baseline**	0.5% (6/1162) ‡ (0.2 to 1.1)		
	**Day 7**	44.4% (292/658) (40.6 to 48.2)	35.3% (162/459) (31.1 to 39.8)	-9.1% (-14.8 to -3.3)
	**Day 28**	16.2% (61/376) (12.8 to 20.3)	8.1% (22/272) (5.4 to 11.9)	-8.1 % (-13.1 to -3.1)
**Serotype 3**	**Baseline**	1.5% (18/1162) (1.5%; 1.0 to 2.4)		
	**Day 7**	1.1% (7/658) (0.5 to 2.2)	0.9% (4/459) (0.3 to 2.2)	-0.2% (-1.4 to 1.2)
	**Day 28**	0.5% (2/376) (0.1 to 1.9)	0.4% (1/272) (0.06 to 2.0)	-0.2% (-1.5 to 1.3)

CI – confidence interval; n/N – number with serotype-specific virus detected/number of samples included in the analysis; ‡ samples confirmed not to be nOPV2 based on a second rRT-PCR assay that identifies sequences specific to nOPV2.

References

1. Guide for surveillance of adverse events of special interest (AESI) during novel oral polio vaccine type 2 (nOPV2) use. Oct 2022. https://polioeradication.org/wp-content/uploads/2022/06/nOPV2-AESI-surveillance.pdf (accessed 10 Oct 2022).
2. Fagerland MW, Lydersen S, Laake P. Recommended tests and confidence intervals for paired binomial proportions. *Statistics in medicine* 2014; 33(16): 2850-75.
3. WHO. WHO child growth standards and the identification of severe acute malnutrition in infants and children. 2011. http://www.who.int/nutgrowthdb/about/introduction/en/.
